# Tumor-associated macrophages promote progression and the Warburg effect via CCL18/NF-kB/VCAM-1 pathway in pancreatic ductal adenocarcinoma

**DOI:** 10.1038/s41419-018-0486-0

**Published:** 2018-04-18

**Authors:** Huilin Ye, Quanbo Zhou, Shangyou Zheng, Guolin Li, Qing Lin, Lusheng Wei, Zhiqiang Fu, Bin Zhang, Yimin Liu, Zhihua Li, Rufu Chen

**Affiliations:** 1 Guangdong Provincial Key Laboratory of Malignant Tumor Epigenetics and Gene Regulation, Sun Yat-sen Memorial Hospital, Sun Yat-sen University, Guangzhou, Guangdong Province China; 2 Department of Pancreatobiliary Surgery, Sun Yat-sen Memorial Hospital, Sun Yat-sen University, Guangzhou, Guangdong Province China; 3 Department of Medical Oncology, Sun Yat-sen Memorial Hospital, Sun Yat-sen University, Guangzhou, Guangdong Province China; 4 Department of Radiotherapy, Sun Yat-sen Memorial Hospital, Sun Yat-sen University, Guangzhou, Guangdong Province China

## Abstract

Tumor-associated macrophages (TAMs) are frequently found near pancreatic cancer cells, but it is uncertain whether they are involved in pancreatic cancer progression and the Warburg effect. Here, we show that CCL18 secreted by TAMs facilitates malignant progression and induced a glycolytic phenotype in pancreatic cancer, partially owing to paracrine induction of VCAM-1 in pancreatic cancer cells. Reciprocally, VCAM-1-induced lactate production from pancreatic cancer cells with enhanced aerobic glycolysis activates macrophages to a TAM-like phenotype, forming a positive feedback loop. VCAM-1 was found to be highly expressed in human pancreatic ductal adenocarcinoma (PDAC) tissues and cell lines, and is associated with disease progression and predicts clinical outcome in PDAC patients. Flow cytometry analysis further demonstrated that VCAM-1 downregulation induced an accumulation of PDAC cells in G0/G1 phase, accompanied by a significant decrease in S phase. Downregulation of VCAM-1 significantly inhibited proliferation, colony formation, migration, and invasion of PDAC cells *in vitro*, whereas the ectopic expression of VCAM-1 had the opposite effect. VCAM-1 on pancreatic cancer cells might tethers THP-1 monocytes to cancer cells via counter–receptor interaction, providing a survival advantage to pancreatic cancer cells that infiltrate leukocyte-rich microenvironments. Furthermore, downregulation of VCAM-1 could repress tumor growth in mouse xenograft models. In particular, our results highlighted the contribution of VCAM-1 to the maintenance of the Warburg effect in PDAC cells. Finally, we investigated the clinical correlations of CCL18 and VCAM-1 in human PDAC specimens. In summary, these findings indicate that the CCL18/PITPNM3/NF-kB/VCAM-1 regulatory network might provide a potential new therapeutic strategy for PDAC.

## Introduction

Pancreatic ductal adenocarcinoma (PDAC) is the main pathological type of pancreatic cancer, and it has a 5-year survival rate of ~ 6%^1,2^. Tumor-associated macrophages (TAMs) are the most abundant immune-related stromal cells, and they provide a favorable milieu for cancer cells^3^. On the basis of their polarization states, macrophages are divided into three types: the unactivated macrophages (M0 macrophages), the classically activated type 1 (M1 macrophages), and the alternatively activated type 2 (M2 macrophages). Differentiated mature TAMs of multiple types of solid tumors have functions and phenotypes that are similar to those of the M2 macrophages^4,5^. However, until now, the biological effects of TAMs on pancreatic cancer progression and metabolic dysregulation remain largely unknown.

Cancer cells metabolize glucose by aerobic glycolysis (the Warburg effect) rather than through the more energetically efficient oxidative phosphorylation, even in the presence of oxygen^6,7^. Accumulating evidence indicates that epigenetic, oncogenic, or anti-oncogenic mutations have an important role in metabolic reprogramming of tumor cells. However, other key players such as tumor environmental factors involved in regulation of pancreatic cancer metabolism and their role in tumor progression are poorly elucidated^8,9^.

In this study, our work provides the valid evidence for reciprocal signaling interactions between TAMs and pancreatic cancer cells, shedding new light on the utilization of CCL18/PITPNM3/NF-kB/VCAM-1 axis as a potential novel therapeutic target for the treatment of pancreatic cancer.

## Results

### Infiltration of CCL18-positive TAMs in pancreatic cancer and characterization of THP-1-derived M2 macrophages

To identify and quantify the amount of infiltrated TAMs with the M2 phenotype, we tested the expression of CCL18, the hallmark of TAMs in paraffin-embedded pancreatic cancer samples by immunohistochemistry (IHC). Our results showed that the expression of CCL18 level in pancreatic cancer was significantly higher than that in corresponding normal pancreatic tissues (Supplementary Figure S1A). It was recently reported that pancreatic TAMs are primarily polarized M2 macrophages that are associated with cancer progression and metastasis^10,11^. Hence, we utilized an experimental model of monocyte-derived macrophage polarization as described in the Supplementary Methods^12^. The morphology of THP-1 cells following exposure to stimuli is shown in Supplementary Figure S1B. Consistently, the expression of CD68 and CD206 as determined by flow cytometry-based analysis significantly increased after differentiation, indicating that the cells became more M2-macrophage-like (Supplementary Figure S1C).

To further confirm the phenotype of the macrophages, we evaluated the mRNA and protein level of several M2-macrophage markers (CD163, CD206, fibronectin, CCL22, CCL18, and interleukin (IL)-10) using quantitative reverse transcriptase-polymerase (qRT-PCR) and enzyme-linked immunosorbent assay (ELISA), respectively. After 24 h of incubation with IL-4 and IL-13, CD206, fibronectin, and IL-10 expression were slightly increased, whereas the expression of CD163, CCL18, and CCL22 was unchanged compared with that of the respective control group. However, if the incubation time was increased to 48 h or even further to 72 h, the mRNA expression level of these six M2-macrophage markers was much higher (Supplementary Figure S1D-I). Consistent with the results described above, the expression pattern of M2 markers was also confirmed at the protein level by ELISA for CCL18, CCL22, and IL-10 (Supplementary Figure S1J-L). In conclusion, these results suggested that IL-4 and IL-13 successfully induced the alternative activated M2 phenotype macrophages.

### TAMs upregulate the expression of VCAM-1 in pancreatic cancer cells

**Fig. 1:**
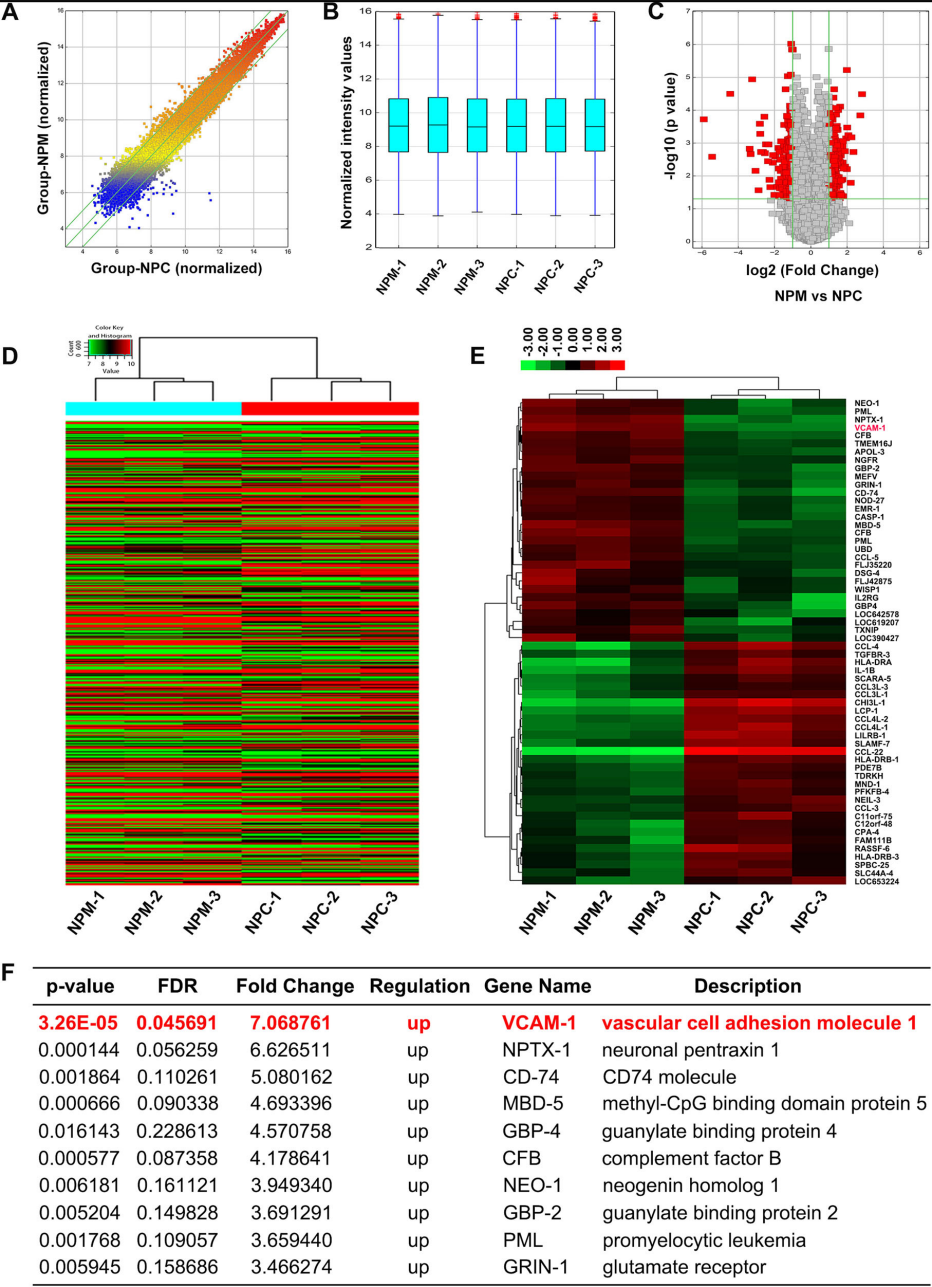
Differences and characterizations in mRNA expression profiles between pancreatic cancer cell line PANC-1-alone control groups (NPC groups) and the PANC-1-co-cultured TAMs groups (NPM groups). **a** Scatter plots are used to evaluate the difference in the expression of mRNAs between the NPC groups and the NPM groups. The values plotted on *X* and *Y* axes are the averaged normalized signal values of each group (log2 scaled). The middle green line refers to no difference between the two groups, and the flanking green lines represent twofold changes. The mRNAs above the top green line and below the bottom green line indicate more than twofold changes between the two groups. **b** Box plots for the normalized gene expression data of the NPC groups and the NPM groups. **c** Volcano plots used for visualizing differential expression between two different conditions. The vertical lines correspond to twofold (log2 scaled) up and down, respectively, and the horizontal line represents a *p* value of 0.05 (−log10 scaled). The red points in plot represent the differentially expressed mRNAs with statistical significance. **d** Hierarchical cluster analysis of all target mRNAs. The mean entities of all target mRNAs, where at least three out of six samples have flags in present or marginal. Flags are attributes that denote the quality of the entities using methods from GeneSpring software. **e** Hierarchical cluster analysis of the top 30 up and downregulated mRNAs. Red and green colors represent up- and downregulated genes, respectively. **f** The top 10 upregulated mRNAs are listed by fold change, among which the cell adhesion molecule VCAM-1 was the most upregulated gene with ~ 7.07-fold change

As TAMs demonstrated potent protumoral properties in pancreatic cancer, we aimed to characterize the downstream molecular events responsible for TAMs-mediated pancreatic cancer malignant progression using the Human 12 × 135 K Gene Expression Array manufactured by Roche NimbleGen. Using a transwell coculture system, we co-cultured PANC-1 pancreatic cancer cells and TAMs for 4 days and subsequently investigated the differential expression profiles of mRNA in PANC-1 cells between the PANC-1-alone control group (NPC groups) and the PANC-1-co-cultured TAMs group (NPM groups). A total of 45,033 mRNA targets were detected by microarray probes in our two groups of samples. The scatter diagram, box, and volcano plot showed all the differentially expressed mRNAs with statistical significance between two experiment groups (Fig. 1a-c). Overall, 498 mRNAs were evaluated to be differentially modulated by fold change > 2.0, *p* value < 0.05 and FDR < 0.05, among which 216 mRNAs were upregulated, whereas 282 mRNAs were downregulated (GEO, http://www.ncbi.nlm.nih.gov/geo/, ID: GSE109110). Heat map analysis and the hierarchical clustering showed that the mRNA expression patterns were distinguishable between these two groups (Fig. 1d,e). In the present study, the top 10 upregulated mRNAs are listed by fold change, among which the cell adhesion molecule VCAM-1 was the most upregulated gene with ~ 7.07-fold change (Fig. 1f).

**Fig. 2:**
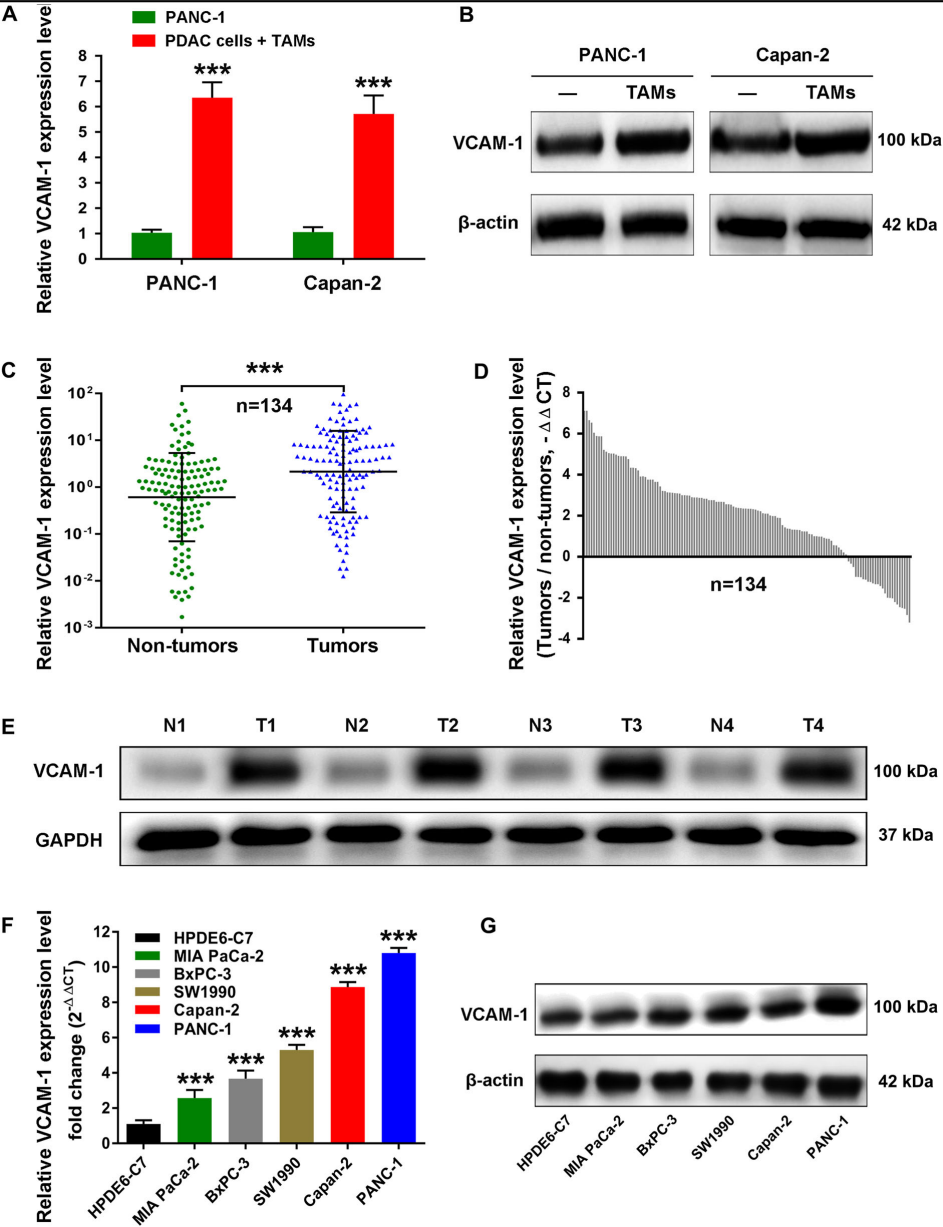
VCAM-1 is aberrantly overexpressed in PDAC tissues and cell lines. **a**,** b** The mRNA and protein levels of VCAM-1 in PANC-1 and Capan-2 cells were measured by qRT-PCR and western blotting analysis. The PANC-1 and Capan-2 cells were cultured alone, or co-cultured with TAMs for 4 days. **c**,** d** The mRNA level of VCAM-1 in 134 paired PDAC tissues and corresponding adjacent non-tumorous tissues by qRT-PCR. VCAM-1 expression levels from all tissues were normalized to β-actin expression (∆CT) and then compared with a non-tumorous tissue and converted to fold change (2^−∆∆CT^). **e** The protein level of VCAM-1 in 4 paired PDAC tissues and their corresponding adjacent non-tumorous tissues by western blotting analysis. **f**,** g** The mRNA and protein levels of VCAM-1 were evaluated in five pancreatic cancer cell lines compared with the pancreatic ductal epithelium cell line HPDE6-C7 by qRT-PCR and western blotting analysis. ***: *p* < 0.001

Aberrant VCAM-1 expression occurs in various solid tumor, including breast tumor, melanomas, and renal carcinoma^13,14^. However, the role of VCAM-1 in pancreatic cancer remains elusive. Hence, we identified VCAM-1 as a gene of interest and set out to determine whether VCAM-1 facilitates malignant progression of pancreatic cancer and participates in the cross-talk between tumor cells and TAMs. RT-qPCR and western blotting showed that VCAM-1 was upregulated in PANC-1 and Capan-2 PDAC cells only when co-cultured with M2-polarized macrophages, validating our microarray results (Fig. 2a, b). To investigate VCAM-1 mRNA expression levels in PDAC, we performed qRT-PCR analysis on total RNA extracted from 134 PDAC tissues and their matched non-neoplastic counterparts. Our current results showed that VCAM-1 mRNA was significantly overexpressed in PDAC samples in comparison with those in corresponding normal tissues (Fig. 2c, d). Subsequently, we randomly selected four paired PDAC samples to evaluate the VCAM-1 protein expression level using western blotting analysis. In agreement with the above-mentioned PCR observations, the results confirmed that VCAM-1 protein level was significantly upregulated in PDAC tissues (Fig. 2e). Moreover, five PDAC cell lines (PANC-1, Capan-2, SW1990, BxPC-3, and MIA PaCa-2) also showed significantly higher VCAM-1 mRNA and protein levels than the pancreatic ductal epithelium cell line HPDE6-C7, with the first two highest expressions observed in PANC-1 and Capan-2 cells (Fig. 2f, g).

### NF-kB activation is involved in CCL18/PITPNM3-induced VCAM-1 upregulation in pancreatic cancer cells

To identify the cytokines secreted by TAMs that upregulated the expression of VCAM-1 of PDAC cells, we first performed qRT-PCR to screen a panel of cytokines related to M2 macrophages in IL-4-treated vs untreated THP-1 monocyte-derived macrophages. As shown in Supplementary Figure S2A, CCL18 is the most abundantly paracrine cytokine in IL-4-treated M2-polarized macrophages compared with IL-4-untreated M0 macrophages. Using qRT-PCR and ELISA analysis, we next examined the CCL18 mRNA and protein level in various PDAC cell lines (PANC-1, Capan-2, BxPC-3, and SW1990). Compared with IL-4-unactivated M0 macrophages, these four PDAC cell lines exhibited faint CCL18 mRNA expression levels. IL-4, which is sufficient to induce the CCL18 expression in THP-1-derived macrophages, did not upregulate the CCL18 mRNA or the soluble CCL18 protein level in the culture supernatant of PDAC cells (Supplementary Figure S2B-C). Our data revealed that CCL18 was either not expressed or was merely expressed at a very low level in cultured PDAC cells.

Next, we measured the CCL18 mRNA levels in fresh frozen PDAC tissues, using normal pancreatic tissues as a control. Using qRT-PCR analysis, we found that the mRNA levels of CCL18 from PDAC tissues were significantly increased compared with those of normal pancreatic tissues (Supplementary Figure S2D). Moreover, ELISA assay further confirmed that the CCL18 protein levels in the serum of patients with PDAC were significantly higher than in those with benign pancreatic disease (Supplementary Figure S2E). Recent studies have reported that CCR6, CCR8, PITPNM3 (also named PYK2 N-terminal domain-interacting receptor 1, Nir1) and GPR30 are potential receptors of CCL18^15,16^. Therefore, we then evaluated the mRNA expression level of these CCL18 potential receptors in PDAC cell lines by qRT-PCR. Our data indicated that these four potential receptors were diversely expressed in different PDAC lines, among which PITPNM3 is the most abundantly expressed receptor in all four cell lines (Supplementary Figure S2F). In addition, using qRT-PCR and IHC, we found that the mRNA and protein levels of PITPNM3 from PDAC tissues were markedly increased compared with normal pancreatic tissues (Supplementary Figure S2G-H). These results suggest TAMs-derived CCL18 may exert effects on the biological behavior of pancreatic cancer cells through binding to the overexpressed receptor PITPNM3.

Previous studies have reported that CCL18 released by TAMs promotes pancreatic cancer growth and metastasis, causing poor survival of PDAC patients^17,18^. Therefore, we examined whether CCL18/PITPNM3 axis regulates the VCAM-1 expression level in PDAC cells. We found that PDAC cells treated with 20 ng/ml CCL18 had a significant increase in the mRNA and the protein levels of VCAM-1. On the other hand, when co-cultured with TAMs and anti-CCL18-neutralizing antibody (10 μg/ml) or when silencing PITPNM3 by siRNA before treating cells with 20 ng/ml CCL18, the effect of CCL18 in upregulating the VCAM-1 expression level was abrogated, as evidenced by the return of VCAM-1 mRNA and protein level to the baseline level (Supplementary Figure S3A-B). However, the mechanism by which CCL18/PITPNM3 axis elevates the expression level of VCAM-1 in PDAC cells remains unclear.

It is reported that multiple regulatory factors were involved in the regulation of VCAM-1 expression, including NF-kB, MAPKK 1/2, PLC, p300/CBP, PKC, EGFR, mTOR, PI3K, and miR-126^19,20^. A recent study showed that CCL18 from activated macrophages induced epithelial–mesenchymal transition of breast cancer cells through the activation of NF-kB pathway^21^. Hence, we proposed that CCL18/PITPNM3 biological axis might upregulate VCAM-1 expression of PDAC cells via activating NF-kB. To clarify the roles of NF-kB in CCL18/PITPNM3-induced VCAM-1 expression, we measured the phosphorylation of IKK, IKBα and p65 using western blotting analysis. As shown in Supplementary Figure S3C-E, CCL18 remarkably increased the VCAM-1 mRNA and protein level, and simultaneously enhanced the level of phospho-IKK, phospho-IKBα, and phospho-p65 in PITPNM3-overexpressing Capan-2 cells. Interestingly, the effect of CCL18 in inducing the VCAM-1 expression and the phosphorylation of IKK, IKBα, and p65 in Capan-2 cells was significantly attenuated when PITPNM3 was silenced by corresponding siRNA. In addition, as expected, the treatments with the p65 specific inhibitor BAY-117082 and the IKBα-specific inhibitor JSH-23 prohibited the effect of CCL18 in inducing VCAM-1 upregulation, as evidenced by the return of VCAM-1 mRNA and protein levels to the baseline levels. As shown in Supplementary Figure S3F, we found that NF-kB activity was escalated to a 3.4-fold increase in CCL18-treated Capan-2 cells. The TNF-α-treated PDAC cells were used as positive control. In line with the results above, we revealed that CCL18-induced p65 nuclear translocation could be reversed by both NF-kB pathway inhibitors (BAY-117082 and JSH-23) and si-PITPNM3, suggesting that CCL18 induced the activation of the NF-kB pathway through the overexpressed receptor PITPNM3 (Supplementary Figure S3G). Collectively, our results indicated that NF-kB activation is involved in the CCL18/PITPNM3-mediated upregulation of VCAM-1 in PDAC cells.

### VCAM-1 mediates proliferation and G1-S checkpoint of PDAC cells *in vitro*

**Fig. 3:**
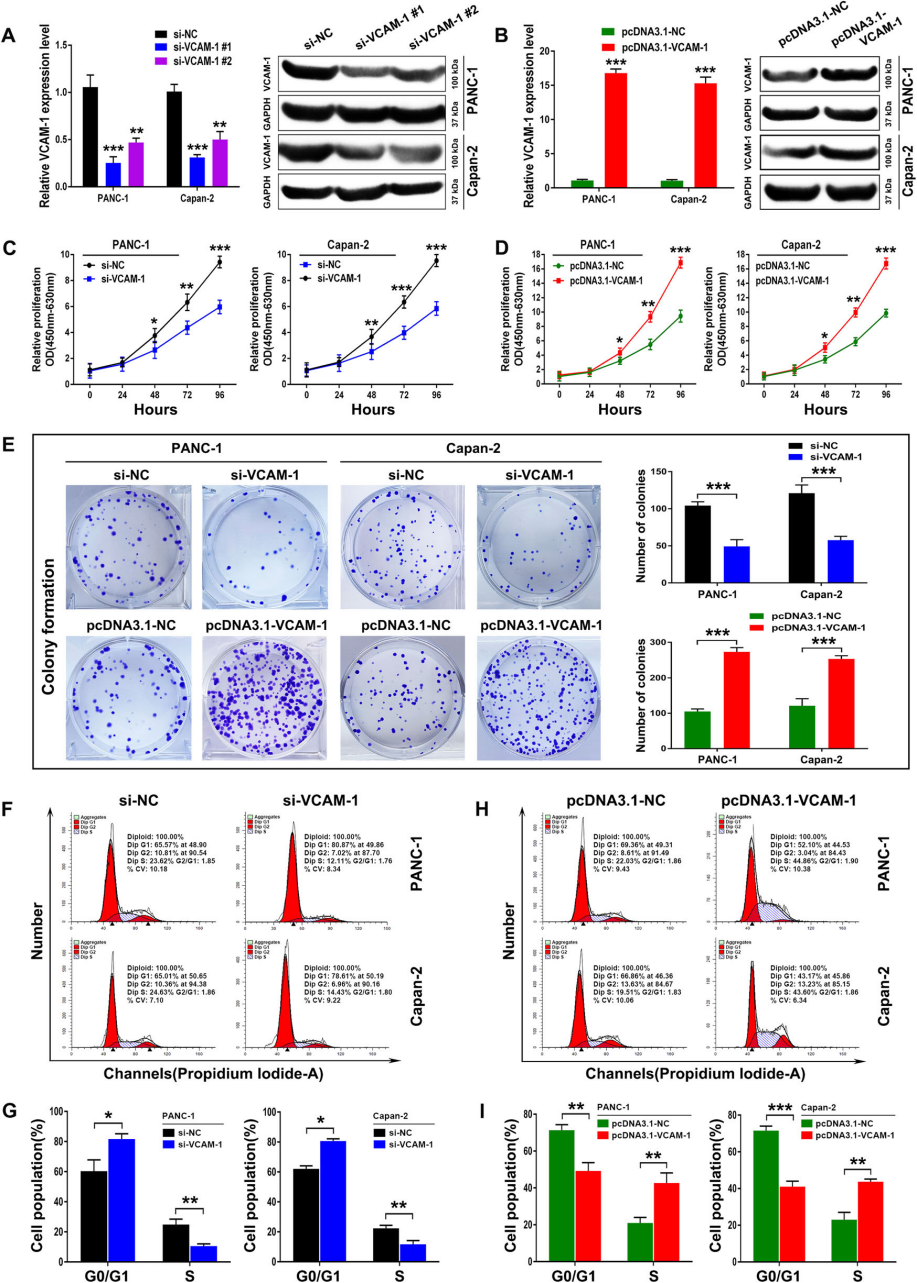
VCAM-1 mediates proliferation and G1-S checkpoint of PDAC cells *in vitro*. **a** The expression of VCAM-1 was suppressed by specific siRNAs in PANC-1 and Capan-2 cells. **b** The expression of VCAM-1 was overexpressed by transfecting pcDNA3.1-VCAM-1 into PANC-1 and Capan-2 cells. **c** The cell viability of si-NC or si-VCAM-1 transfected PANC-1 and Capan-2 cells, as determined by CCK-8 assay. **d** The cell viability of pcDNA3.1-NC or pcDNA3.1-VCAM-1 transfected PANC-1 and Capan-2 cells, as determined by CCK-8 assay. (**e**) Top: The proliferation of si-NC or si-VCAM-1 transfected PANC-1 and Capan-2 cells by colony formation assay. Bottom: The proliferation of pcDNA3.1-NC or pcDNA3.1-VCAM-1 transfected PANC-1 and Capan-2 cells by colony formation assay. **f**, **g** The cell cycle distribution of si-NC or si-VCAM-1 transfected PANC-1 and Capan-2 cells by flow cytometry analysis. **h**, **i** The cell cycle distribution of pcDNA3.1-NC or pcDNA3.1-VCAM-1 transfected PANC-1 and Capan-2 cells by flow cytometry analysis. Values represented the mean ± SD from three independent experiments. *, ** or ***: significantly different from the corresponding control, *p* < 0.05, *p* < 0.01 or *p* < 0.001, respectively, by Student’s *t*-test

To further investigate the causal role of VCAM-1 in PDAC progression, *in vitro* functional characterizations were performed. First, qRT-PCR and western blotting analysis revealed that the expression of VCAM-1 was significantly reduced by specific siRNAs and upregulated by pcDNA3.1-cDNA vectors in PANC-1 and Capan-2 cells, correspondingly (Fig. 3a, b). Compared with si-NC cells, VCAM-1 downregulation greatly attenuated tumor cell proliferation in PANC-1 and Capan-2 cells, as determined by a CCK-8 assay. In contrast, VCAM-1 overexpression had the opposite effect (Fig. 3c, d). Consistently, the colony formation assays further confirmed these findings (Fig. 3e). In addition, flow cytometry analysis further demonstrated that VCAM-1 downregulation induced an accumulation of PDAC cells in G0/G1 phase, accompanied by a significant decrease in cells in S phase (Fig. 3f, g). In contrast, VCAM-1 overexpression had the opposite effect on apoptosis and cell cycle distribution (Fig. 3h, i). However, dysregulation of VCAM-1 made no difference to the proportion of apoptotic cells (data not shown). Thus, the VCAM-1-induced promotion of PDAC cells proliferation appeared to be mediated by modulation of the G1-S checkpoint, rather than by apoptosis.

### VCAM-1 promotes PDAC cell migration and invasion *in vitro*

**Fig. 4:**
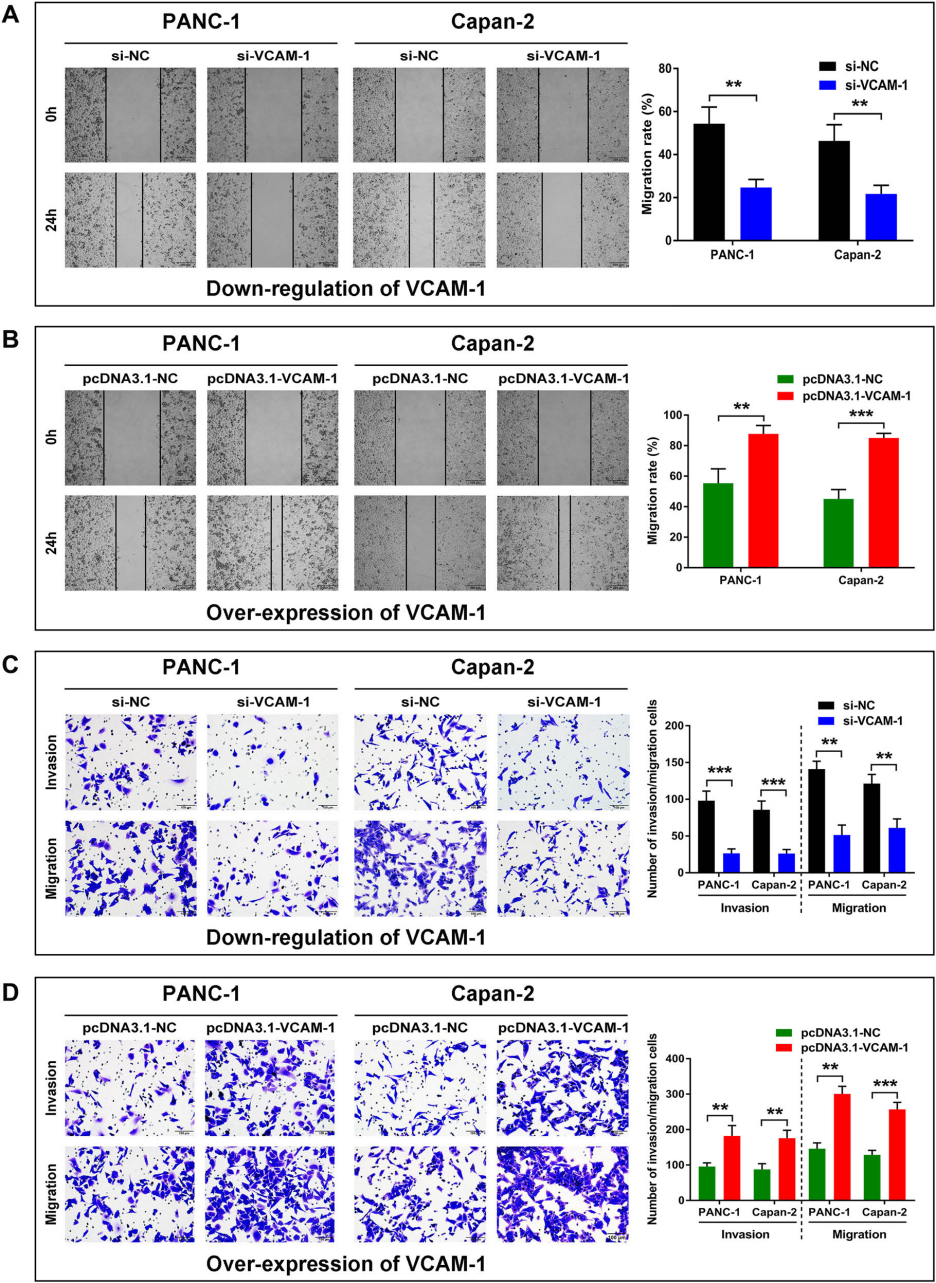
VCAM-1 mediates migration and invasion of PDAC cells *in vitro*. PDAC Cell line PANC-1 and Capan-2 were treated as in described in the Materials and methods. **a** The motility of PANC-1 and Capan-2 cells transfected with si-VCAM-1 when compared with the controls by wound-healing assay. **b** The motility of PANC-1 and Capan-2 cells transfected with pcDNA3.1-VCAM-1 when compared with the controls by wound-healing assay. **c** The migration and invasion of PANC-1 and Capan-2 cells transfected with si-VCAM-1 when compared with the controls by transwell assay. **d** The migration and invasion of PANC-1 and Capan-2 cells transfected with pcDNA3.1-VCAM-1 when compared with the controls by transwell assay. Values represented the mean ± SD from three independent experiments. *, ** or ***: significantly different from the corresponding control, *p* < 0.05, *p* < 0.01 or *p* < 0.001, respectively, by Student’s *t*-test

Enhanced cell migration and invasion abilities have a pivotal role in cancer development, leading to poor prognosis. Our wound-healing scratch assay illustrated that VCAM-1 knockdown in PDAC cells markedly decreased cell motility compared with the si-NC control group, whereas the ectopic expression of VCAM-1 had the opposite effect (Fig. 4a, b). Moreover, the transwell assay further demonstrated that knockdown of VCAM-1 dramatically attenuated the migration and invasion of PANC-1 and Capan-2 cells (Fig. 4c). Conversely, the overexpression of VCAM-1 significantly increased the migration and invasion of these two cell lines (Fig. 4d). These observations indicated that VCAM-1 facilitated PDAC cell migration and invasion *in vitro*.

### VCAM-1 mediates recruitment of TAMs to pancreatic cancer cells *in vitro*

Monocytic cells including macrophages enhance tumor progression. Macrophage recruitment seems to correlate with multiple molecular mechanisms such as specific chemokines and endothelial–leukocyte adhesion molecules^22^. To gain insights into the pathological cross-talk between tumor and stromal cells, we next explored the expression of VCAM-1 in PDAC cells and its dynamic relationship with macrophages recruitment. To determine whether VCAM-1 on PDAC cells can bind leukocytes, we incubated Hoechst 33342-labeled PANC-1 or Capan-2 monolayers with a suspension of BCECF-AM-labeled THP-1 cells, a human monocyte cell line. Our cell–cell binding assays demonstrated that the si-NC PANC-1 or Capan-2 cells bound over four- to sixfold as many THP-1 cells as did the si-VCAM-1 PDAC cells (Supplementary Figure S4A-C). Moreover, addition of a blocking antibody against VCAM-1 significantly inhibited the binding of THP-1 cells to PDAC cells (Supplementary Figure S4D-F). Thus, we concluded that VCAM-1 on PDAC cells might tether monocytes to cancer cells via counter–receptor interaction, providing a survival advantage to PDAC cells that infiltrate leukocyte-rich microenvironments.

### VCAM-1 facilitates the Warburg effect of PDAC cells *in vitro*

**Fig. 5:**
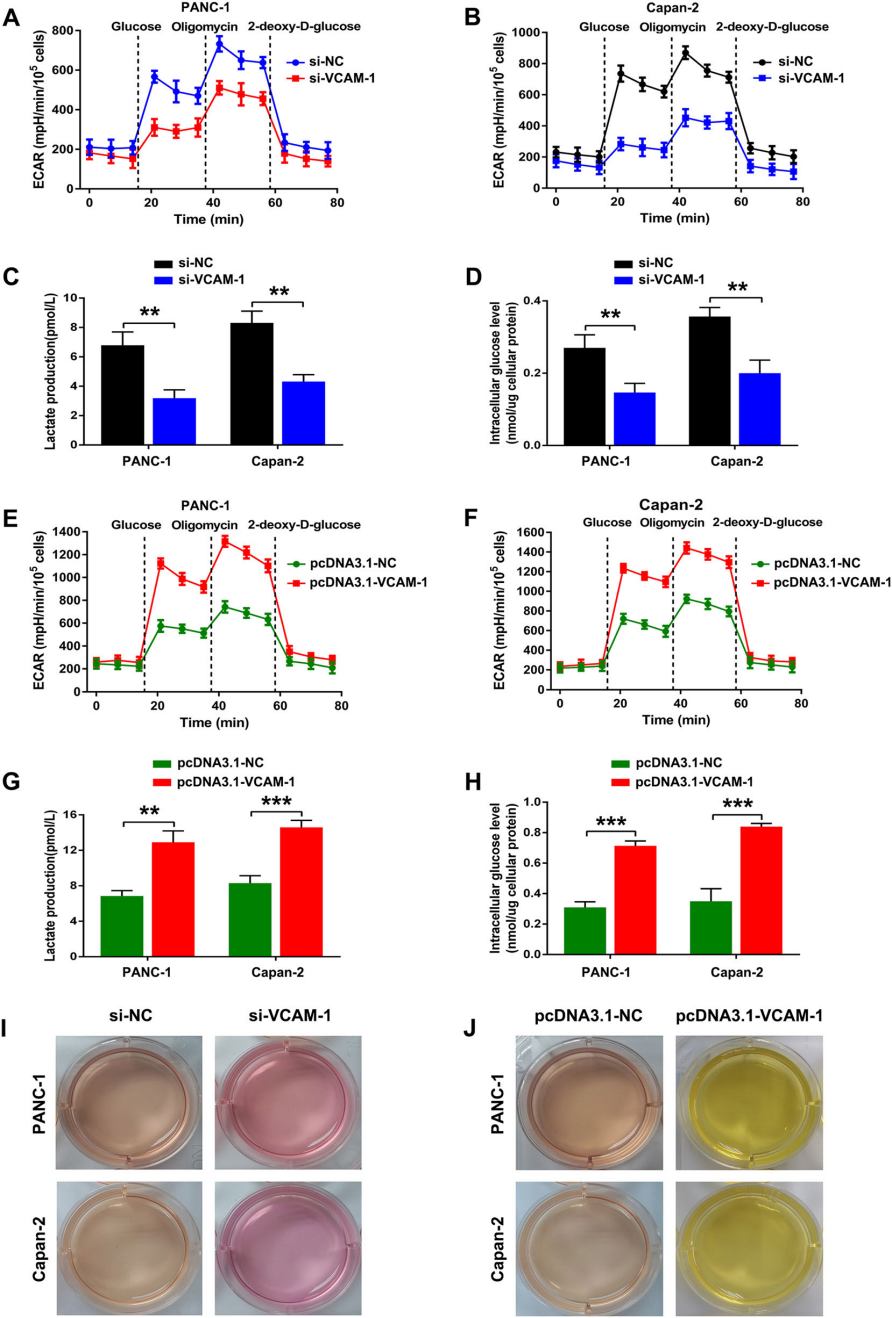
VCAM-1 modulates the Warburg effect of PDAC cells *in vitro*. **a**,** b** Silencing VCAM-1 expression abrogated the glycolytic capacity of PANC-1 and Capan-2 cells, as reflected by ECAR analysis. **c**,** d** Silencing VCAM-1 expression inhibited the glucose uptake and lactate production of PANC-1 and Capan-2 cells. Intracellular glucose levels were measured and normalized based on protein concentration. **e**, **f** Ectopic VCAM-1 expression facilitated the glycolytic capacity of PANC-1 and Capan-2 cells, as reflected by ECAR analysis. **g**, **h** Ectopic VCAM-1 expression promoted the glucose uptake and lactate production of PANC-1 and Capan-2 cells. **i** PANC-1 and Capan-2 cells expressing si-NC or si-VCAM-1 were cultured under normoxic conditions for 24 h. Acidification of the culture medium was evaluated by visually inspecting the color of the medium. **j** PANC-1 and Capan-2 cells expressing pcDNA3.1-NC or pcDNA3.1-VCAM-1 were cultured under normoxic conditions for 24 h. Acidification of the culture medium was evaluated by visually inspecting the color of the medium. ** or ***: significantly different from the corresponding control, *p* < 0.01 or *p* < 0.001, respectively, by Student’s *t*-test

Some of the most recent studies have validated that breast cancer cells with aberrant expression of VCAM-1 have a growth advantage in leukocyte-rich milieu via excessive activation of the PI3K/Akt biological axis^23^. Accumulating evidence has confirmed that PI3K/Akt pathway hyperactivation is closely connected with various cellular processes that support the survival and growth of tumor cells, including the so-called “Warburg effect”^24^. Thus, we examined the change in aerobic glycolysis in VCAM-1-silenced PANC-1 and Capan-2 cells by using Seahorse XF analyzers. Our data revealed that the extracellular acidification rate (ECAR) significantly decreased in VCAM-1 knockdown cells, indicating that silencing VCAM-1 inhibited the glycolytic process in PDAC cells (Fig. 5a, b). In contrast, VCAM-1 overexpression had the opposite effect (Fig. 5e, f). Accordingly, silencing VCAM-1 also led to a strong decrease in glucose uptake and lactate production (Fig. 5c, d), whereas VCAM-1 overexpression resulted in a significant increase in glucose uptake and lactate production of both PDAC cell lines (Fig. 5g, h). Consistent with the above results, the acidification of the culture medium via visually inspecting the color of the medium further confirmed these findings (Fig. 5I, j). Collectively, our results reinforced the contribution of adhesion molecule VCAM-1 to the maintenance of the Warburg effect in PDAC cells.

### Lactate produced by VCAM-1-mediated Warburg effect regulates the functional polarization of macrophages *in vitro*

**Fig. 6:**
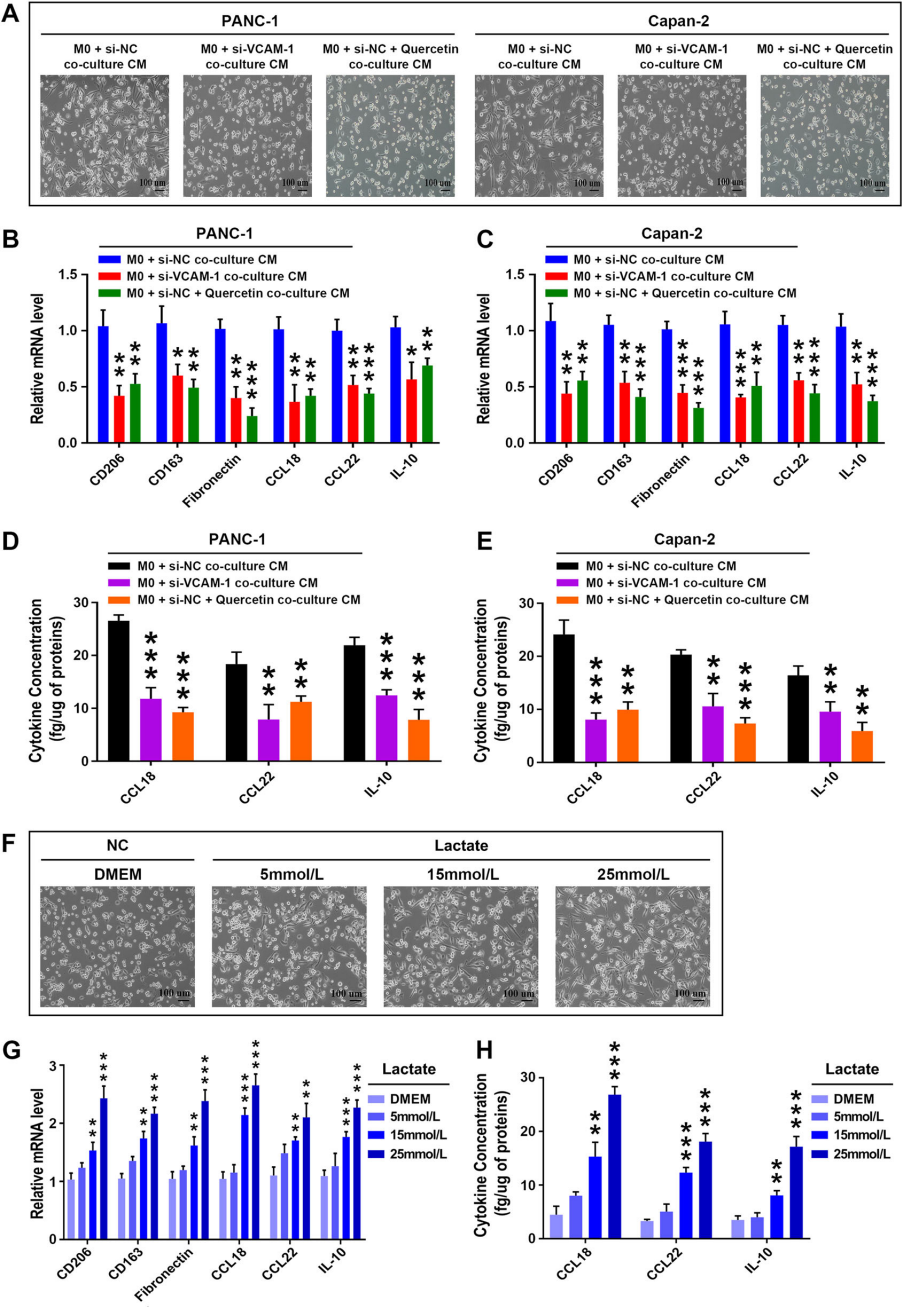
Lactate regulates the functional polarization of macrophages *in vitro*. **a** M0 macrophages incubated with conditioned medium (CM) derived from si-VCAM-1 PDAC cells or treated with quercetin alone were less spindle-shaped and were rounder in appearance compared with M0 macrophages incubated with conditioned medium derived from si-NC PDAC cells. **b**–**e** Compared with M0 macrophages incubated with conditioned medium derived from si-NC PDAC cells, M0 macrophages incubated with conditioned medium derived from si-VCAM-1 PDAC cells or treated with quercetin alone had significantly lower mRNA levels of CD206, CD163, fibronectin, CCL18, CCL22, and IL-10 **b**,** c** and showed markedly lower protein levels of CCL18, CCL22, and IL-10 **d**, **e**, as determined by qRT-PCR and ELISA assay, respectively. **f**–**h** VCAM-1-derived lactate facilitates the M2-like polarization of macrophages in a dose-dependent manner. **f** Lactate stimulation induced the alternative activated M2 phenotype macrophages as characterized by a morphological change from an epithelial-like, cobblestone phenotype to a more spindle-shaped mesenchymal phenotype. **g**,** h** Compared with M0 macrophages incubated with 0 mmol/l lactate, M0 macrophages treated with increasing concentrations (5, 15, 25 mmol/l) of lactate had significantly higher mRNA levels of CD206, CD163, fibronectin, CCL18, CCL22, and IL-10 and showed prominently higher protein levels of CCL18, CCL22 and IL-10, as determined by qRT-PCR and ELISA assay, respectively

Malignant conversion of normal cells accompanied by aerobic glycolysis results in enhanced glucose uptake and lactate production^6^. However, whether lactate contribute to the phenotype polarization of macrophages in the tumor microenvironment of PDAC is yet to be fully explored. To further investigate the biological effect of VCAM-1-derived lactate on the phenotype and function of macrophages, we incubated THP-1-derived unactivated macrophages (M0) with conditioned medium derived from si-NC or si-VCAM-1 PDAC cells combined with quercetin. As shown in Fig. 6a, M0 macrophages incubated with conditioned medium derived from si-VCAM-1 PDAC cells or treated with quercetin alone were less spindle-shaped and were rounder in appearance, and thus morphologically distinct from M0 macrophages incubated with conditioned medium derived from si-NC PDAC cells. Accordingly, compared with M0 macrophages incubated with conditioned medium derived from si-NC PDAC cells, M0 macrophages incubated with conditioned medium derived from si-VCAM-1 PDAC cells or treated with quercetin alone had significantly lower mRNA level of CD206, CD163, fibronectin, CCL18, CCL22, and IL-10 and showed a markedly lower protein levels of CCL18, CCL22, and IL-10 (Fig. 6b–e). As quercetin is a naturally occurring flavonoid and has been demonstrated to inhibit MCT-1 (monocarboxylate transporter-1) and MCT-2-mediated lactate uptake, we focused on the possibility that tumor-derived lactate is the soluble factor responsible for the polarization of macrophages^25^. To investigate whether lactate alone is sufficient to regulate the functional differentiation of macrophages, we treated M0 macrophages with different concentrations (0, 5, 15, 25 mmol/l) of lactate *in vitro*. Lactate stimulation induced the alternative activated M2 phenotype macrophages as identified by a morphological variation from a cobblestone phenotype to a more mesenchymal spindle-shaped phenotype (Fig. 6f). Moreover, compared with M0 macrophages incubated with 0 mmol/l lactate (Dulbecco's Modified Eagle Medium (DMEM) medium alone), M0 macrophages treated with increasing concentrations (5, 15, 25 mmol/l) of lactate had a significant higher mRNA level of CD206, CD163, fibronectin, CCL18, CCL22, IL-10 and showed a prominent higher protein level of CCL18, CCL22, and IL-10 (Fig. 6g, h). Taken together, these data indicated that VCAM-1-derived lactate facilitates the M2-like polarization of macrophages in a dose-dependent manner.

### VCAM-1 is important for tumor growth *in vivo*

**Fig. 7:**
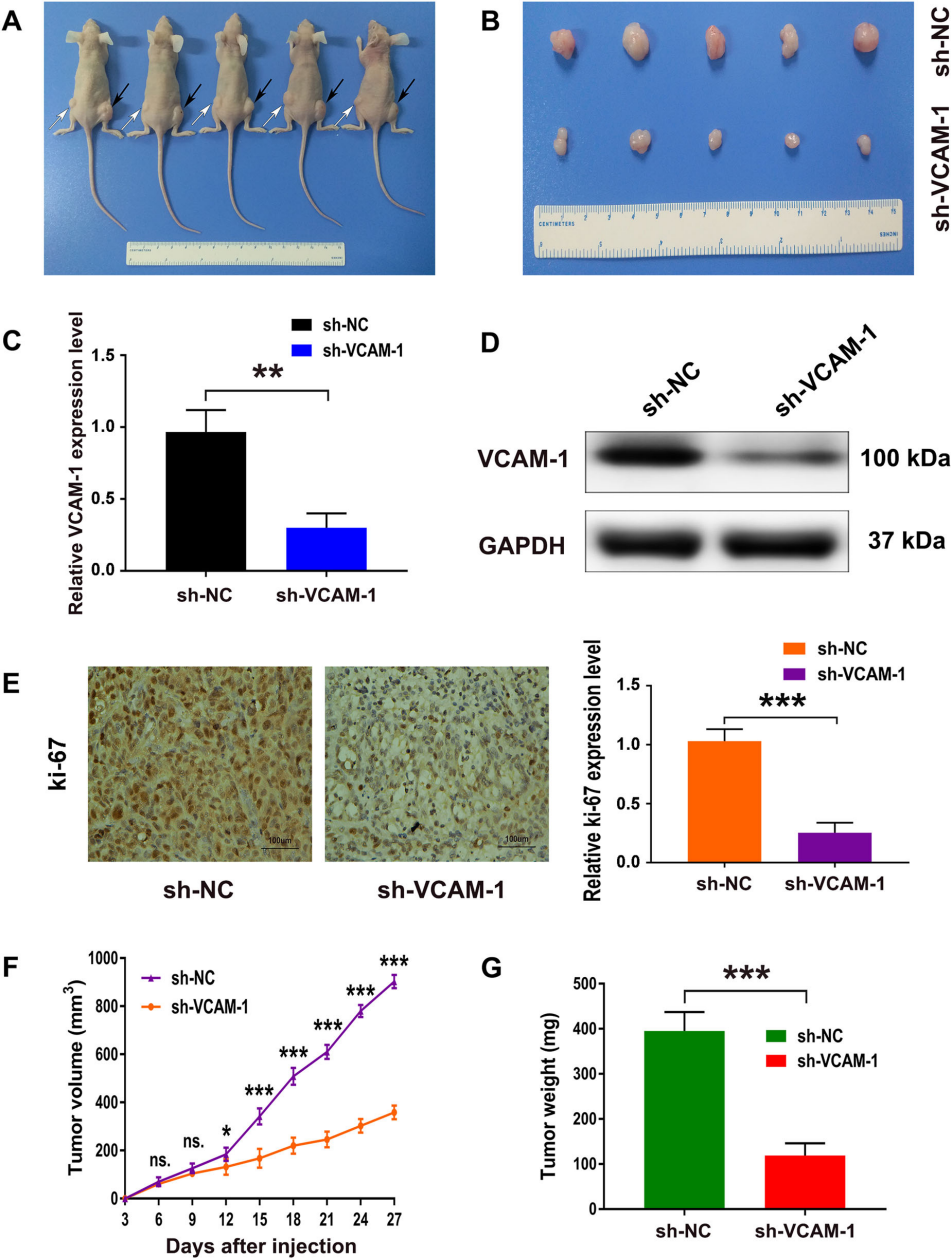
Knockdown of VCAM-1 significantly inhibits tumor growth in mouse xenograft models. **a**, **b** A subcutaneous injection of PANC-1 cells with stable knockdown of VCAM-1 or mock cells was administered into the subcutaneous bilateral hind leg of nude mice. At 28 days after subcutaneous injection, PANC-1 cells transfected with sh-VCAM-1 (white arrow) and mock cells (black arrow) produced primary tumors and a representative figure of the tumors formed is shown. **c**, **d** The qRT-PCR and western blot analyzed the mRNA and protein levels of VCAM-1 in tumor tissues from sh- VCAM-1 PANC-1 cells compared with sh-NC PANC-1 cells. **e** Representative images (magnification: × 200) of IHC staining of the tumor. The IHC staining showed that VCAM-1 knockdown decreased the proliferation index Ki-67. **f** Tumor growth curve. The points indicate mean (*n* = 5), and the bars indicate the SD. **g** Tumor weights are shown as the means of tumor weights ± SD when the tumors were harvested. ns.: not significantly different. *, ** or ***: significantly different from the corresponding control, *p* < 0.05, *p* < 0.01 or *p* < 0.001, respectively, by Student’s *t*-test

To investigate the role of VCAM-1 on tumor formation *in vivo*, we administered a subcutaneous injection of PANC-1 cells with stable knockdown of VCAM-1 (sh-VCAM-1) or mock cells (sh-NC) into the subcutaneous bilateral hind leg of athymic nude mice. All mice developed xenograft tumors at the injection site (Fig. 7a, b). Using qRT-PCR and western blotting analysis, we confirmed the inhibition of VCAM-1 in the xenotransplanted tumors from the sh-VCAM-1 group (Fig. 7c,d). Our data demonstrated that tumors from the sh-VCAM-1 group showed a decreased positive rate of Ki-67 compared with the sh-NC group (Fig. 7e). Consistent with the *in vitro* results, xenograft tumors grown from VCAM-1-silenced PANC-1 cells had smaller mean size and weight than xenograft tumors grown from mock cells (Fig. 7f,g). Taken together, these findings validated that VCAM-1 has a crucial role in PDAC proliferation capacity in mouse xenograft models.

### CCL18 and VCAM-1 overexpression correlates with clinicopathological characteristics and prognosis of PDAC patients

**Fig. 8:**
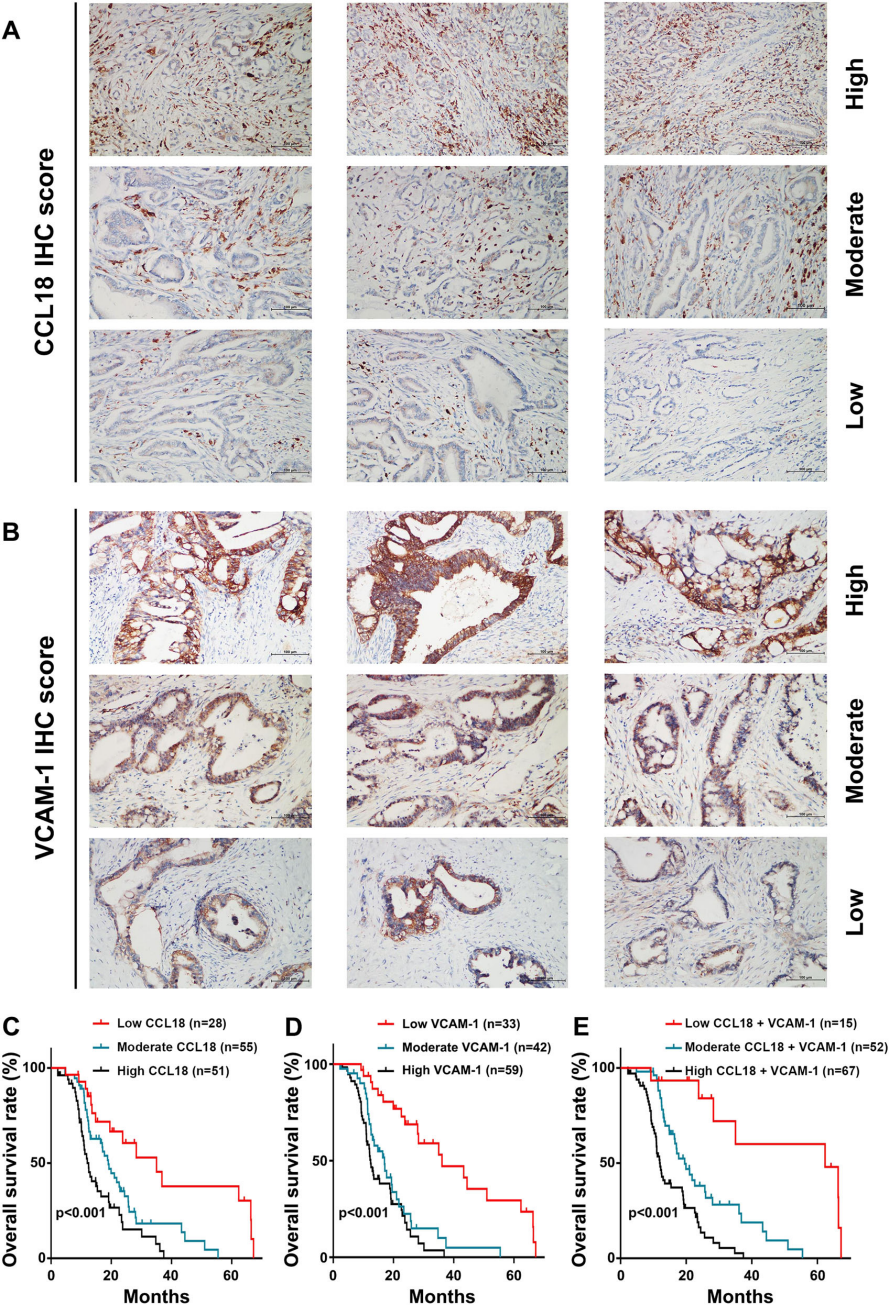
CCL18 and VCAM-1 overexpression is associated with clinicopathological characteristics and poor prognosis of PDAC patients. The expression levels of VCAM-1 and CCL18 were scored semiquantitatively based on staining intensity and distribution using the immunoreactive score (IRS) as described in the Supplementary Methods. **a** Representative images of CCL18 staining in PDAC tissues (categorization: low, moderate, and high). **b** Representative images of VCAM-1 staining in PDAC tissues (categorization: low, moderate, and high). **c** The Kaplan–Meier analysis of overall survival stratified by the immunoreactive score of CCL18 expression. **d** The Kaplan–Meier analysis of overall survival stratified by the immunoreactive score of VCAM-1 expression. The log-rank test was used to compare differences between groups. **e** The survival analysis showed that a higher combined CCL18 and VCAM-1 immunoreactive score correlated with poorer prognosis in PDAC patients (log-rank test, *p* *<* 0.001)

To investigate the clinical relevance of the CCL18/VCAM-1 axis in PDAC, we measured the expression levels of CCL18 and VCAM-1 protein in 134 paraffin-embedded human PDAC samples by IHC. The representative images of three groups were shown in Fig. 8a, b. We also analyzed the relationship between the intensity of CCL18 or VCAM-1 staining and clinicopathological features. Statistical analysis confirmed that CCL18 overexpression was only correlated with the TNM stage, whereas VCAM-1 overexpression was correlated with the TNM stage, lymph node metastasis, and perineural invasion (Table 1). Furthermore, the survival analysis showed that higher CCL18 or VCAM-1 staining intensity correlated with poorer prognosis in PDAC patients (log-rank test, *p* *<* 0.001, Fig. 8c, d). In addition, the survival analysis showed that higher combined CCL18 and VCAM-1 staining intensities correlated with poorer prognosis in PDAC patients (log-rank test, *p* *<* 0.001, Fig. 8e). Moreover, the univariate and multivariate analysis demonstrated that the TNM stage, lymph node metastasis, VCAM-1 expression, and CCL18 expression were independent prognostic factors (Table 2).

**Table 1:** Correlation between VCAM-1 and CCL18 expression and clinicopathologic characteristics of PDAC patients

Characteristics	N of cases	VCAM-1 level				CCL18 level			
		L	M	H	*p* value	L	M	H	*p* value
Total cases	134	33	42	59		28	55	51	
Gender					0.619				0.990
Male	77	21	22	34		16	32	29	
Female	57	12	20	25		12	23	22	
Age					0.767				0.979
<60	59	14	17	28		12	24	23	
≥60	75	19	25	31		16	31	28	
Differentiation					0.448				0.169
Well	22	4	5	13		1	9	12	
Moderate	86	24	29	33		22	33	31	
Poor	26	5	8	13		5	13	8	
TNM stage (AJCC)^b^					0.009**				0.036*
I	35	16	10	9		12	15	8	
II	36	7	15	14		9	16	11	
III	41	7	9	25		4	18	19	
IV	22	3	8	11		3	6	13	
Lymph node metastasis					0.004**				0.275
Negative	56	22	15	19		14	25	17	
Positive	78	11	27	40		14	30	34	
Perineural invasion					0.013*				0.482
Negative	45	18	12	15		11	20	14	
Positive	89	15	30	44		17	35	37	

^a^*χ*^2^-test, *p < 0.05, **p < 0.01.

^b^American Joint Committee on Cancer (AJCC), patients were staged in accordance with the 7th Edition of the AJCC Cancer’s’ TNM Classification; *N of cases* number of cases; *T stage* tumor stage; *TNM* tumor node metastasis; *L* low; *M* moderate; *H* high

**Table 2:** Univariate and multivariate analysis of overall survival in PDAC patients (*n* = 134)

Variables	Characteristics	Univariate analysis			Multivariate analysis		
		HR	95% CI	*p* value	HR	95% CI	*p* value
Age	<60 (ref)						
	≥60	0.920	0.615–1.378	0.687			
Gender	Female (ref)						
	Male	0.948	0.629–1.428	0.797			
Differentiation	Well (ref)						
	Moderate	0.951	0.537–1.685	0.863			
	Poor	1.701	0.868–3.335	0.122			
TNM stage	I (ref)						
	II	1.491	0.853–2.606	0.161	0.994	0.531–1.859	0.984
	III	2.596	1.458–4.621	0.001**	1.823	0.963–3.449	0.065
	IV	3.976	2.064–7.659	<0.001***	2.252	1.100–4.609	0.026*
Lymph node metastasis	Negative (ref)						
	Positive	2.601	1.640–4.123	<0.001***	1.950	1.193–3.187	0.008**
Perineural invasion	Negative (ref)						
	Positive	1.543	0.976–2.438	0.063			
VCAM-1 expression	Low (ref)						
	Moderate	3.680	1.965–6.891	<0.001***	2.332	1.152–4.772	0.019*
	High	5.448	2.930–10.130	<0.001***	2.948	1.445–6.014	0.003**
CCL18 expression	Low (ref)						
	Moderate	2.585	1.340–4.985	0.005**	1.721	0.839–3.532	0.139
	High	4.523	2.296–8.910	<0.001***	2.267	1.080–4.762	0.031*

*HR* hazard ratio; *95% CI* 95% confidence interval; *TNM* tumor node metastasis; *T stage* tumor stage; *ref* reference

Cox regression analysis, *p < 0.05, **p *<* 0.01, ***p *<* 0.001

## Discussion

The tumor microenvironment has recently been proven to contain an autocrine–paracrine communication circuit that reinforces cancer proliferation and metastasis through reciprocal signaling^26,27^. TAMs around the cancer regions usually exert their tumor-supporting functions by the secretion of cytokines and inflammatory mediators^16,17^. Our results confirmed that the expression level of CCL18 in pancreatic cancer was significantly higher than that in corresponding normal pancreatic tissues and that CCL18 is the most abundantly expressed cytokine in IL-4-activated M2 macrophages. In addition, our data revealed that TAMs-derived CCL18 specifically binds to PITPNM3 at the cellular membrane and subsequently upregulates VCAM-1 expression in PDAC cells by activating NF-kB signal transduction.

Previous studies indicated that VCAM-1 was overexpressed in various solid tumors^13,14,23^. However, little is known about the clinical and biological function of VCAM-1 in pancreatic cancer thus far. As reported in our work, we examined the aberrant expression of VCAM-1, its association with clinicopathological characteristics and patients’ prognosis, and its biological effects on pancreatic cancer *in vitro* and *in vivo*. More interestingly, our cell–cell binding assays demonstrated that aberrant expression of VCAM-1 on PDAC cells might tethers monocytes to cancer cells through counter–receptor interactions, providing a survival advantage to PDAC cells that infiltrate leukocyte-rich microenvironments. However, the molecular mechanism of VCAM-1 on cancer cell biological behaviors deserves further research.

Thus far, the mechanistic link between the adhesion molecule VCAM-1 and cellular glucose metabolism in PDAC cells still remains largely unknown. As exhibited in our results, silencing VCAM-1 inhibited the glycolytic process, glucose uptake and lactate production in PDAC cells. Tumor-derived lactate has been considered to function as a vital paracrine molecule to regulate the biological characteristics of the neighboring stromal cells^28^. In this study, we found that VCAM-1-derived lactate can facilitate the M2-like polarization of macrophages in a dose-dependent manner, indicating a reciprocal feedback loop between CCL18-positive TAMs and PDAC cells.

In conclusion, we demonstrated that CCL18-positive TAMs were important in malignant progression and induced a glycolytic phenotype in pancreatic cancer, partially owing to paracrine induction of VCAM-1 in PDAC cells. This TAM-related Warburg effect in pancreatic cancer was critical for the maintenance of TAMs-like features, thus forming a regulatory feedback loop within tumor microenvironment. Our study provides the first connection between CCL18/PITPNM3/NF-kB/VCAM-1 axis and PDAC progression and the Warburg effect, raising the possibility of new options for clinical interventions for pancreatic cancer patients.

## Materials and methods

### Patients and clinical samples

Between 2008 and 2016, we collected 134 paired PDAC and non-cancerous tissues from the surgical patients in our department. No patient received anticancer treatment before surgery. The Hospital’s Protection of Human Subjects Committee approved our protocol, and all the patients were informed before surgery. All samples were evaluated and histologically diagnosed with PDAC by pathologic examination with hematoxylin–eosin staining. None of these patients received any preoperative chemotherapy or radiotherapy. All samples were quickly frozen in liquid nitrogen and transferred to a −80 °C freezer for later use. The detailed clinical and pathological features of patients are shown in Table 1. The follow-up data were collected completely, and overall survival (OS) was explicitly defined as the time interval beginning at the date of surgery and ending at the date of death or at the end of follow-up (October 2016).

### Human gene expression array analysis

The metadata spreadsheet, matrix table, raw data files, and microarray platform were submitted to the Gene Expression Omnibus database repository at the National Center for Biotechnology Information (GEO, http://www.ncbi.nlm.nih.gov/geo/, ID: GSE109110). Human Gene Expression Array analysis was performed as described in the Supplementary Methods.

### Cell culture

Human PDAC cell lines (PANC-1, Capan-2, SW1990, BxPC-3, and MIA PaCa-2), the immortal human pancreatic duct epithelial cell line (HPDE6-C7) and the human THP-1 monocytes were purchased from the American Type Culture Collection (USA). Detailed procedure for differentiation and polarization of THP-1 cells and the details of other procedures could be seen in the Supplementary Methods.

### RNA extraction and qRT-PCR

Total RNA was extracted from the paired pancreatic cancer tissues and non-cancerous tissues or cultured cells by TRIzol reagent (Invitrogen, USA). The details of the next procedure were described in the Supplementary Methods. All primer sequences for the qRT-PCR assays are supplied in Table S1 (see Additional file 1). For the relative gene expressions in tissues or cells, the values were at first normalized to β-actin levels as ΔCt and then transferred to the fold change (2^−ΔΔCt^) by comparing them with a control that was chosen from one of the samples.

### Cell transfection and viral infection

All the small interfering RNAs (siRNAs) been used at the study were as follows: PITPNM3 siRNA (si-PITPNM3), VCAM-1 siRNA (si-VCAM-1), and scrambled siRNA (si-NC) were gained from GenePharma Co. (Shanghai, China). The short hairpin RNA (sh-RNA) interference was used at the stable suppression of VCAM-1. All oligonucleotide sequences were listed in Table S2 (see Additional file 2). The details of transfection and infection procedures are described in the Supplementary Methods.

### Expression construct

The sequence of VCAM-1 was synthesized and subcloned into pcDNA3.1 (Invitrogen, Shanghai, China). Ectopic expression of VCAM-1 was achieved by using the pcDNA3.1-VCAM-1 transfection, and empty pcDNA vector (pcDNA3.1-NC) was used as control. The expression level of VCAM-1 was detected by qRT-PCR and western blotting analysis.

### Western blotting analysis

Western blotting assay was performed as described in the Supplementary Methods. Primary antibodies were rabbit anti-human VCAM-1 antibody (1:1000, #ab134047, Abcam), rabbit anti-human t-p65 antibody (1:800, #8242, CST, Boston, USA), rabbit anti-human p-p65 antibody (1:800, #3033, CST, Boston, USA), rabbit anti-human IKKα antibody (1:1000, #2682, CST, Boston, USA), rabbit anti-human p-IKK antibody (1:1000, #2697, CST, Boston, USA), rabbit anti-human IKBα antibody (1:800, #4814, CST, Boston, USA), rabbit anti-human p-IKBα antibody (1:800, #2859, CST, Boston, USA), rabbit anti-human β-actin antibody (1:2000, #ab8227, Abcam) or rabbit anti-human GAPDH antibody (1:2000, #ab18162, Abcam). GAPDH or β-actin was used as a loading control. Horseradish peroxidase-linked secondary antibody is goat anti-rabbit IgG (1: 5000; CST, Boston, USA).

### IHC analysis

Paraffin-embedded samples of primary carcinomas were immunostained for primary rabbit anti-human VCAM-1 antibody (1:200, #ab134047, Abcam), rabbit anti-human CCL18 antibody (1:30, MAB394, R&D), rabbit anti-human PITPNM3 antibody (1:50, Santa Cruz Biotechnology, Santa Cruz, CA), and Ki-67 (1:500, #ab6526, Abcam). The expression levels of VCAM-1, CCL18, PITPNM3 and Ki-67 were scored according to the staining intensity and proportion by the immunoreactive score as described in the Supplementary Methods. Staining was professionally assessed by two pathologists based on the scoring criteria. These procedures are described in the Supplementary Methods.

### Tumor formation assay

The athymic Balb/c nude mice aged from 4–6 weeks were used for the *in vivo* tumorigenesis experiments. Tumor volume was calculated as follow: volume = (L × W^2^) / 2 (V, volume; L, length diameter; W, width diameter). All the necessary steps were taken to minimize the suffering and distress caused to the mice. The procedures of tumor formation assay are described in the Supplementary Methods.

### Cell binding assay

THP-1 cells were labeled with 0.1 μg/ml BCECF/AM (green) for 1 h at 37 °C and were washed twice with growth medium. Pancreatic cancer cells (2.5 × 10^4^ cells/well) were seeded in 24-well culture plates one day in advance. Next, the BCECF/AM-labeled THP-1 cells (2.5 × 10^4^ cells/ml) were gently added to the monolayer of PDAC cells after the conditioned medium was removed. After incubation for 1 h at 37 °C, the wells were gently washed twice with warm medium in order to remove non-adherent cells. Hoechst 33342 (blue) was used to stain the nuclei of both cell types. At last, all cells were observed and photographed under a fluorescence microscope.

### Luciferase reporter assay

The pNF-kB-Luc, pTAL-Luc, pRL-TK vectors (Promega Madison, WI, USA) were transfected into cells for 24 h, using Lipofectamine 2000 (Invitrogen, Carlsbad, CA) according to the manufacturer’s instruction, followed by 20 ng/ml rCCL18 or 10ng/ml TNF-α treatment for 12 h. Then, 48 h after transfection, the luciferase activity was assayed by the Dual-luciferase reporter assay system (Promega). The firefly luciferase activity was normalized to the Renilla luciferase activity for all samples.

### Glucose uptake and lactate production assay

Under normoxic conditions, PDAC cells were cultured in DMEM (glucose-free) for 16 h and were then incubated with DMEM (high-glucose) for 24 h. After removing the culture medium, the intracellular glucose levels were then measured by a fluorescence-based glucose assay kit (BioVision, Exton, USA) based on manufacturer’s instructions. Using a lactate oxidase-based colorimetric assay (Beyotime, Wuxi, China), lactate levels were measured according to the manufacturer’s instructions. The cells were plated in a 24-well plate at the density of 2 × 10^5^ cells/ml, and aliquots of media from each well were assessed 24 h later for the amount of lactate present.

### ECAR

Using an XF^e^96 Extracellular Flux Analyzer (Seahorse Bioscience, North Billerica, MA, USA), the extracellular acidification rates were measured according to the manufacturer’s instructions. In brief, 5 × 10^4^ cells were seeded into XF^e^96 culture plates in quadruplicate one day in advance. ECAR was then measured under basal conditions and after sequential treatment of the PDAC cells with glucose, oligomycin and 2-deoxy-D-glucose. The cells were trypsinized and counted for normalization purposes.

### Statistical analysis

The SPSS Statistics 18.0 was used in our statistical analyses. For the analysis of parametric variables, Student’s *t*-test or one-way analysis of variance was used (two-tailed), whereas for non-parametric variables, the chi-square test (*χ*^2^-test) were use. All data were presented as the mean ± SD from at least three independent experiments, unless otherwise noted. The Kaplan–Meier method was used for the assessment of differences in patient survival and the log-rank test was used for a univariate analysis. Univariate and multivariate Cox regression analyses were applied to evaluate the relative risk for each factor. All tests were two-sided, and results with *p* < 0.05 were considered statistically significant.

## Electronic supplementary material

Figure S1

Figure S2

Figure S3

Figure S4

Table S1

Table S2

Supplementary Methods

Supplementary figure legends
