# Supplementary figures and images for: Tumor-associated macrophages promote progression and the Warburg effect via CCL18/NF-kB/VCAM-1 pathway in pancreatic ductal adenocarcinoma
Source: Cell Death Dis. 2018 Apr 18;9(5):453. doi: 10.1038/s41419-018-0486-0 (PMC5906621; doi:10.1038/s41419-018-0486-0)

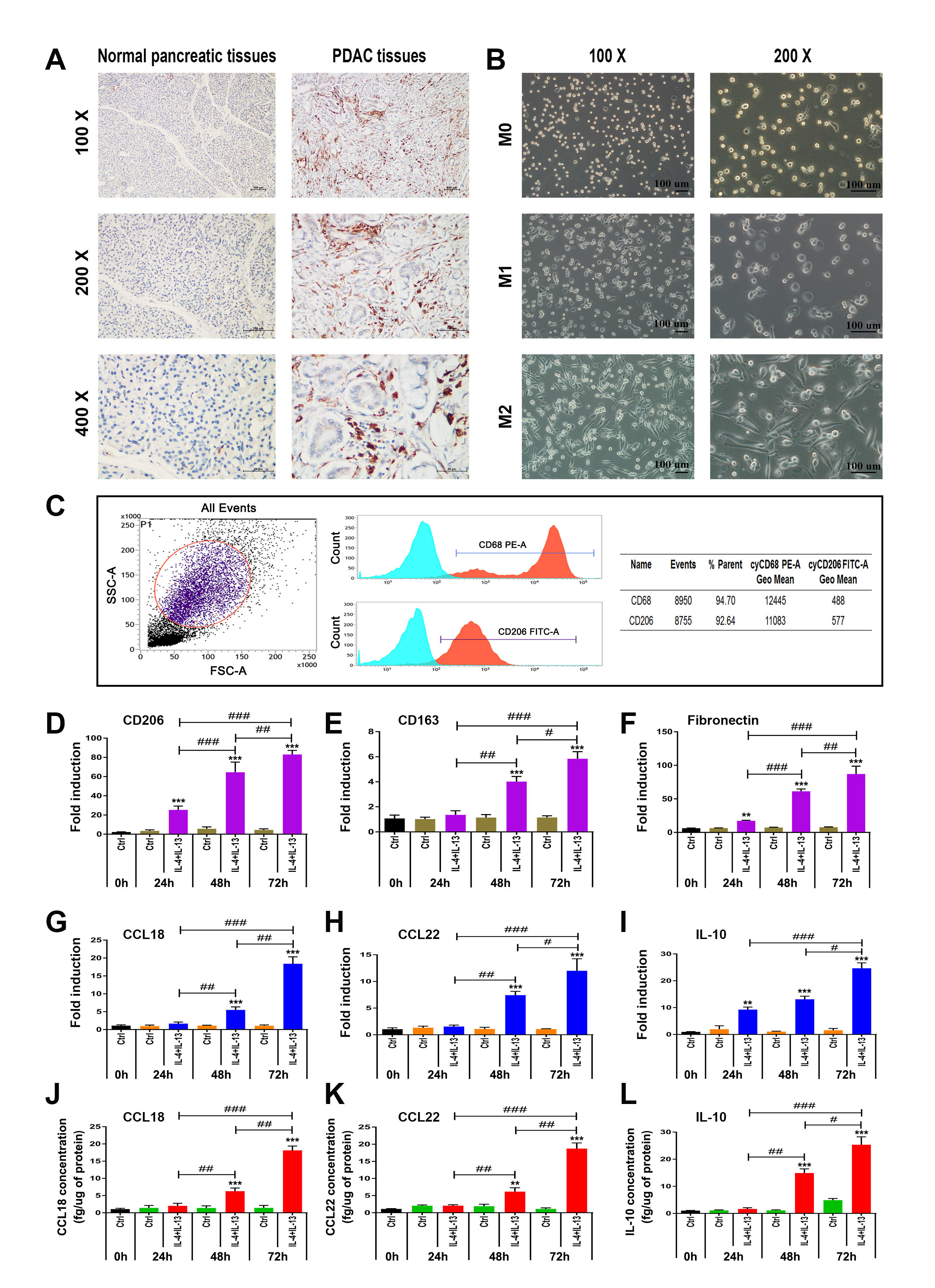

Supplement: Supplementary file 1 — Figure S1 [file 41419_2018_486_MOESM1_ESM.jpg]

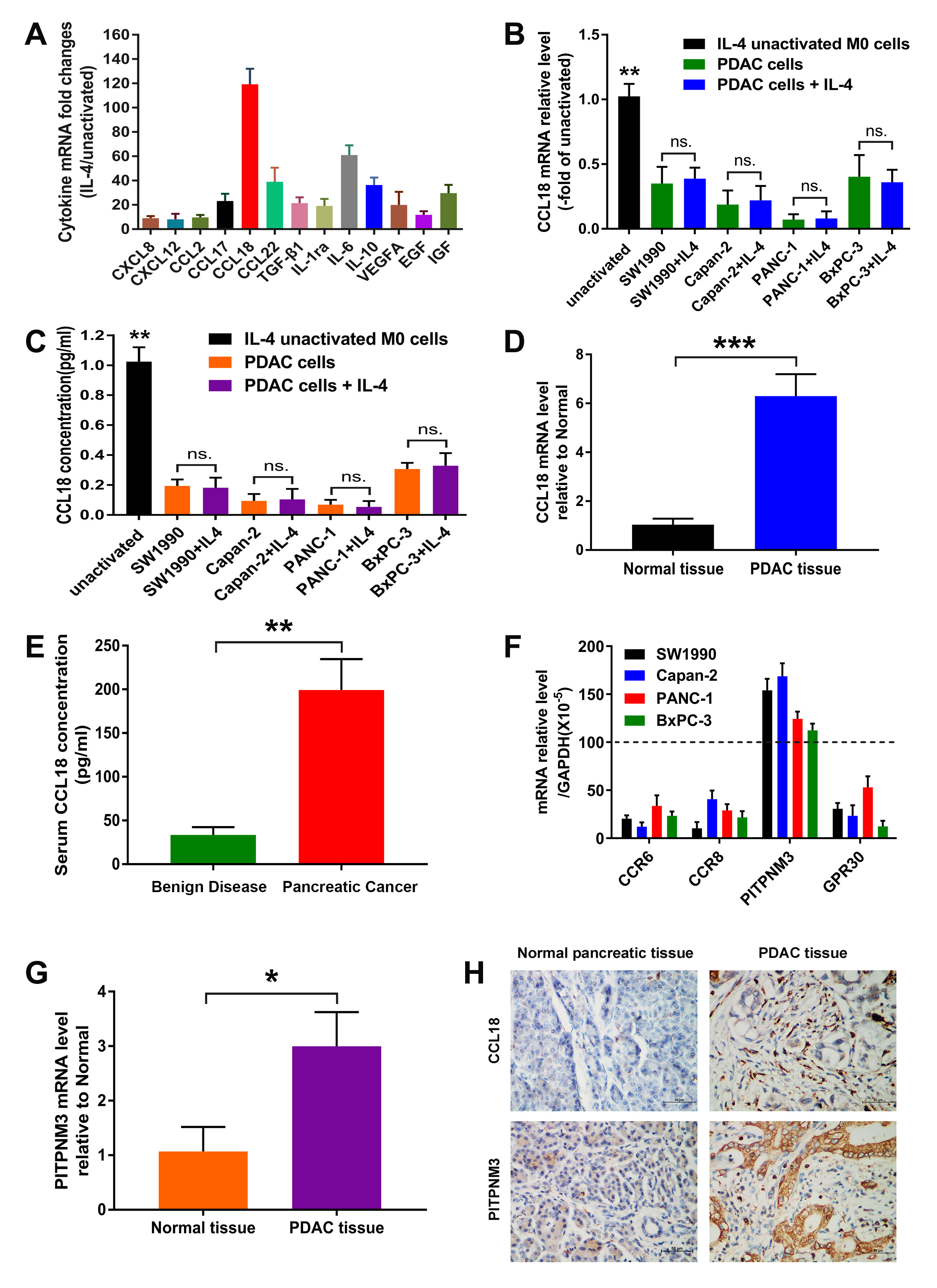

Supplement: Supplementary file 2 — Figure S2 [file 41419_2018_486_MOESM2_ESM.jpg]

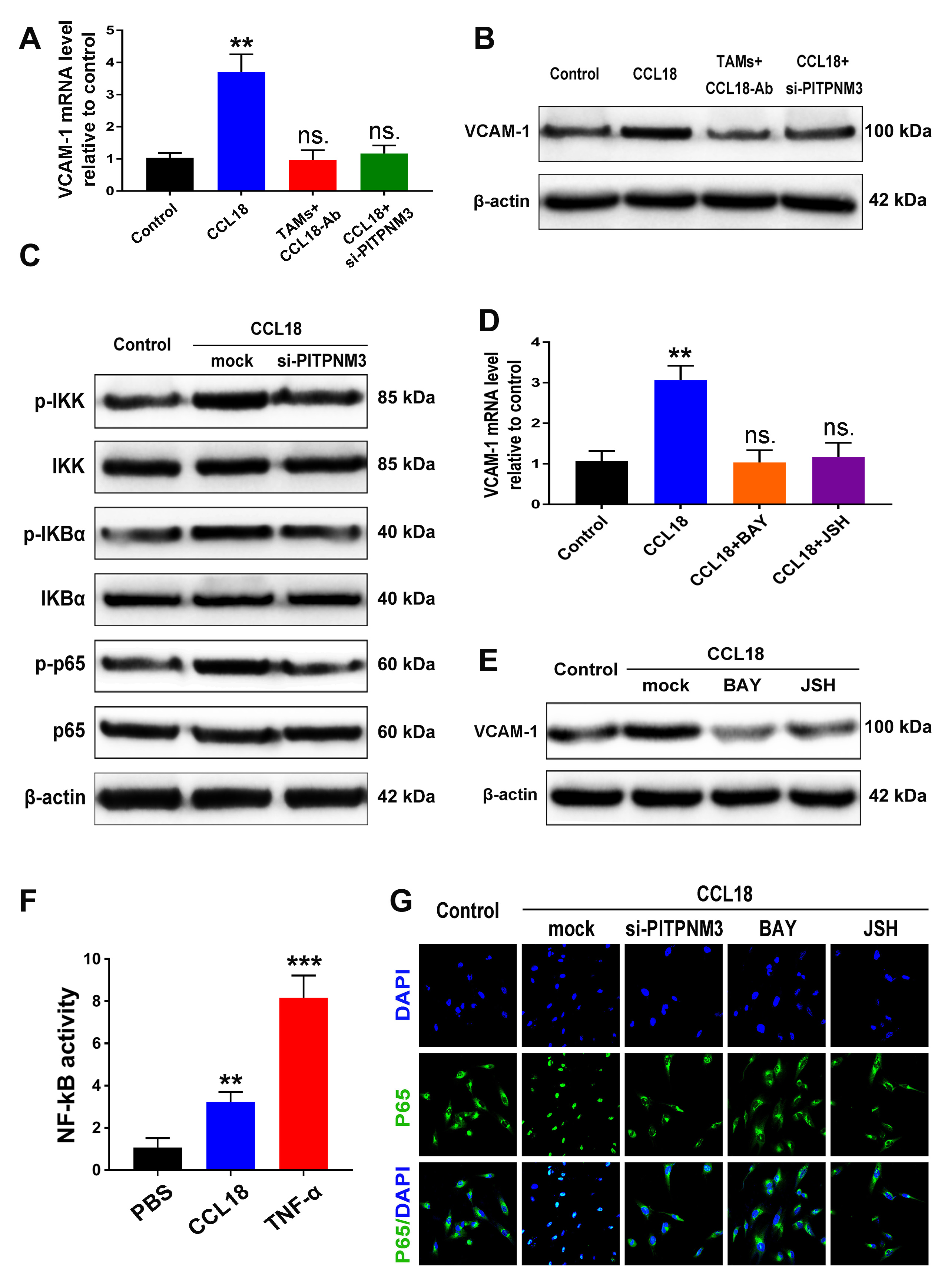

Supplement: Supplementary file 3 — Figure S3 [file 41419_2018_486_MOESM3_ESM.jpg]

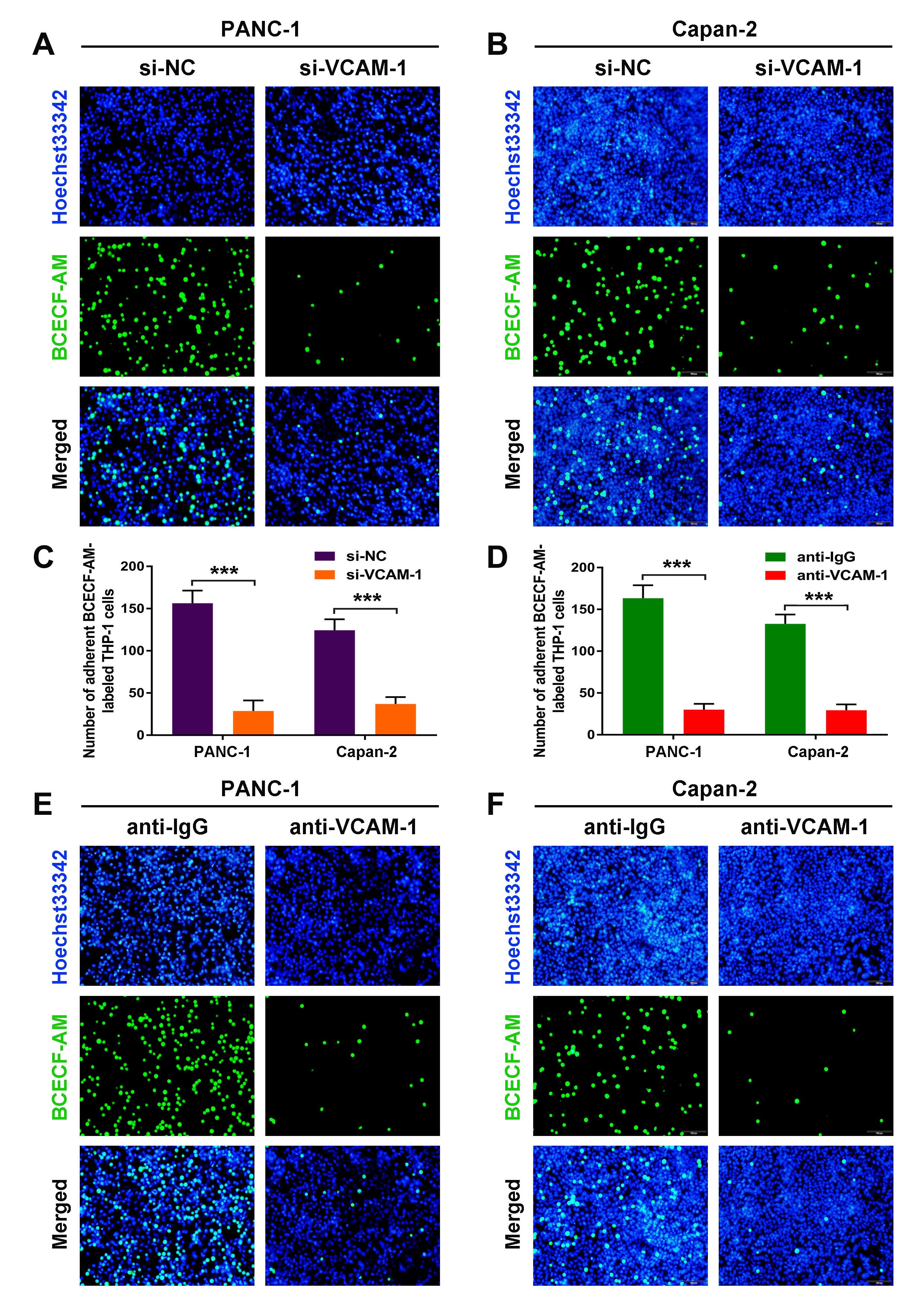

Supplement: Supplementary file 4 — Figure S4 [file 41419_2018_486_MOESM4_ESM.jpg]
