# Supplementary material for: Tumor-associated macrophages promote progression and the Warburg effect via CCL18/NF-kB/VCAM-1 pathway in pancreatic ductal adenocarcinoma
Source: Cell Death Dis. 2018 Apr 18;9(5):453. doi: 10.1038/s41419-018-0486-0 (PMC5906621; doi:10.1038/s41419-018-0486-0)
Supplement: Supplementary file 5 — Table S1 [file 41419_2018_486_MOESM5_ESM.docx]

**Table S1. Primers for qRT-PCR.**

| **Gene Name** | **Direction*** | **Sequence (5’ to 3’)** |
| --- | --- | --- |
| CXCL8 | F | TTTTGCCAAGGAGTGCTAAAGA |
|  | R | AACCCTCTGCACCCAGTTTTC |
| CXCL12 | F | CCCGAAGCTAAAGTGGATTC |
|  | R | TTCAGAGCTGGGCTCCTACT |
| CCL2 | F | AAGATCTCAGTGCAGAGGCTCG |
|  | R | CACAGATCTCCTTGGCCACAA |
| CCL17 | F  R | AGGGACCTGCACACAGAGAC  CTCGAGCTGCGTGGATGTGC |
| CCL18  CCL22  IL-1ra  IL-6  IL-10  TGF-β  EGF  IGF-1  VEGFA  CD206  CD163  Fibronectin  CCR6  CCR8  PITPNM3  GPR30  VCAM-1  β-actin  GAPDH | F  R  F  R  F  R  F  R  F  R  F  R  F  R  F  R  F  R  F  R  F  R  F  R  F  R  F  R  F  R  F  R  F  R  F  R  F  R | CTCTGCTGCCTCGTCTATACCT  CTTGGTTAGGAGGATGACACCT  ATGGCTCGCCTACAGACTGCACTC  CACGGCAGCAGACGCTGTCTTCCA  TCTGTTCTTGGGAATCCATGG  TCAGTGATGTTAACTGCCTCCAG  AATAACCACCCCTGACCCAAC  ACATTTGCCGAAGAGCCCT  AACAAGAGCAAGGCCGTGG  GAAGATGTCAAACTCACTCATGGC  AAGGACCTCGGCTGGAAGTGC  CCGGGTTATGCTGGTTGTA  CTTGTCATGCTGCTCCTCCTG  TGCGACTCCTCACATCTCTGC  GCTCTTCAGTTCGTGTGTGGA  GCCTCCTTAGATCACAGCTCC  ATGACGAGGGCCTGGAGTGTG  CCTATGTGCTGGCCTTGGTGAG  TCCGGGTGCTGTTCTCCTA  CCAGTCTGTTTTTGATGGCACT  TTTGTCAACTTGAGTCCCTTCAC  TCCCGCTACACTTGTTTTCAC  CGGTGGCTGTCAGTCAAAG  AAACCTCGGCTTCCTCCATAA  TTCAGCGATGTTTTCGACTCC  GCAATCGGTACAAATAGCCTGG  GTGTGACAACAGTGACCGACT  CTTCTTGCAGACCACAAGGAC  TCGCTTGTCTCTCACCTGAAC  CAGGAACTCTCTGTAGACCTGG  CACCAGCAGTACGTGATCGG  CATCTTCTCGCGGAAGCTGAT  GGGAAGATGGTCGTGATCCTT  TCTGGGGTGGTCTCGATTTTA  CATGTACGTTGCTATCCAGGC  CTCCTTAATGTCACGCACGAT  ATCACCATCTTCCAGGAGCGA  CCTTCTCCATGGTGGTGAAGAC |

*** F: forward; R: reverse.**
