# Supplementary material for: Tumor-associated macrophages promote progression and the Warburg effect via CCL18/NF-kB/VCAM-1 pathway in pancreatic ductal adenocarcinoma
Source: Cell Death Dis. 2018 Apr 18;9(5):453. doi: 10.1038/s41419-018-0486-0 (PMC5906621; doi:10.1038/s41419-018-0486-0)
Supplement: Supplementary file 6 — Table S2 [file 41419_2018_486_MOESM6_ESM.docx]

**Table S2. Oligonucleotide sequences for this study.**

| **Name** | **Direction*** | **Sequence (5’ to 3’)** |
| --- | --- | --- |
| **si-RNA** |  |  |
| si-PITPNM3 #1 | F  R | CGAAGGACAUCUCUGUCUATT  UAGACAGAGAUGUCCUUCGTT |
| si-PITPNM3 #2 | F | GGAAGACAGAGGAAAGAAATT |
|  | R | UUUCUUUCCUCUGUCUUCCTT |
| si-VCAM-1 #1 | F | GGAGUAAGAAAUUAGAUAATT |
|  | R | UUAUCUAAUUUCUUACUCCTT |
| si-VCAM-1 #2 | F | GGAGAUAGACUUACUGAAATT |
|  | R | UUUCAGUAAGUCUAUCUCCTT |
| si-NC  **sh-RNA**  sh-VCAM-1 #1  sh-VCAM-1 #2  sh-NC | F  R  F  R  F  R  F  R | UUCUCCGAACGUGUCACGUTT  ACGUGACACGUUCGGAGAATT  CCGGAAGGAGTAAGAAATTAGATAACTCGAGTTATCTAATTTCTTACTCCTTTTTTTG  AATTCAAAAAAAGGAGTAAGAAATTAGATAACTCGAGTTATCTAATTTCTTACTCCTT  CCGGAAGGAGATAGACTTACTGAAACTCGAGTTTCAGTAAGTCTATCTCCTTTTTTTG  AATTCAAAAAAAGGAGATAGACTTACTGAAACTCGAGTTTCAGTAAGTCTATCTCCTT  CCGGTTCTCCGAACGTGTCACGTAACTCGAGTTACGTGACACGTTCGGAGAATTTTTG  AATTCAAAAATTCTCCGAACGTGTCACGTAACTCGAGTTACGTGACACGTTCGGAGAA |

*** F: forward; R: reverse.**
