# Supplementary material for: Tumor-associated macrophages promote progression and the Warburg effect via CCL18/NF-kB/VCAM-1 pathway in pancreatic ductal adenocarcinoma
Source: Cell Death Dis. 2018 Apr 18;9(5):453. doi: 10.1038/s41419-018-0486-0 (PMC5906621; doi:10.1038/s41419-018-0486-0)
Supplement: Supplementary file 7 — Supplementary Methods [file 41419_2018_486_MOESM7_ESM.docx]

**Supplementary Methods**

**Human Gene Expression Array analysis**

RNA quantity and quality were measured by NanoDrop ND-1000. RNA integrity was assessed by standard denaturing agarose gel electrophoresis. The Human 12 × 135K Gene Expression Array was manufactured by Roche NimbleGen. This array can simultaneously hybridize 12 samples on each slide. 45033 genes are collected from the authoritative data source including NCBI. Double-strand cDNA (ds-cDNA) was synthesized from total RNA using an Invitrogen SuperScript ds-cDNA synthesis kit in the presence of 100 pmol oligo dT primers. ds-cDNA was cleaned and labeled in accordance with the NimbleGen Gene Expression Analysis protocol (NimbleGen Systems, Inc., USA). Briefly, ds-cDNA was incubated with 4 μg RNase A at 37 °C for 10 minutes and cleaned using phenol: chloroform: isoamyl alcohol, followed by ice-cold absolute ethanol precipitation. The purified cDNA was quantified using a NanoDrop ND-1000. For Cy3 labeling of cDNA, the NimbleGen One-Color DNA labeling kit was used according to the manufacturer’s guideline detailed in the Gene Expression Analysis protocol (NimbleGen Systems, Inc., Madison, WI, USA). One μg ds-cDNA was incubated for 10 minutes at 98 °C with 1 OD of Cy3-9mer primer. Then, 100 pmol of deoxynucleoside triphosphates and 100U of the Klenow fragment (New England Biolabs, USA) were added and the mix was incubated at 37 °C for 2 hours. The reaction was stopped by adding 0.1 volume of 0.5 M EDTA, and the labeled ds-cDNA was purified by isopropanol / ethanol precipitation. Microarrays were hybridized at 42 °C during 16 to 20 hours with 4 μg of Cy3 labeled ds-cDNA in NimbleGen hybridization buffer/hybridization component A in a hybridization chamber (Hybridization System-NimbleGen Systems, Inc., Madison, WI, USA). Following hybridization, washing was performed using the NimbleGen Wash Buffer kit (NimbleGen Systems, Inc., Madison, WI, USA). After being washed in an ozone-free environment, the arrays were scanned by the Axon GenePix 4000B scanner. Slides were scanned at 5 μm/pixel resolution using an Axon GenePix 4000B scanner (Molecular Devices Corporation) piloted by GenePix Pro 6.0 software (Axon). Scanned images (TIFF format) were then imported into NimbleScan software (version 2.5) for grid alignment and expression data analysis. Expression data were normalized through quantile normalization and the Robust Multichip Average (RMA) algorithm included in the NimbleScan software. The Probe level (*_norm_RMA.pair) files and Gene level (*_RMA.calls) files were generated after normalization. All gene level files were imported into Agilent GeneSpring GX software (version 11.5.1) for further analysis. Differentially expressed genes between two groups were identified through Volcano Plot filtering. Hierarchical clustering was performed using the Agilent GeneSpring GX software (version 11.5.1). GO analysis and Pathway analysis were performed using the standard enrichment computation method.

The metadata spreadsheet, matrix table, raw data files and microarray platform were submitted to the Gene Expression Omnibus database repository at the National Center for Biotechnology Information (GEO, http://www.ncbi.nlm.nih.gov/geo/, ID: GSE109110).

**Cell culture**

Human PDAC cell lines (PANC-1, Capan-2, SW1990, BxPC-3 and MIA PaCa-2), the immortal human pancreatic duct epithelial cell line (HPDE6-C7) and the human THP-1 monocytes were purchased from the American Type Culture Collection (ATCC, Manassas, USA). Cells were maintained in Dulbecco’s modified Eagle’s medium (DMEM; GIBCO-BRL; Invitrogen, CA, USA) or Roswell Park Memorial Institute medium (RPMI 1640; GIBCO-BRL; Invitrogen, CA, USA) supplemented with 10% fetal bovine serum (FBS; GIBCO-BRL; Invitrogen, CA, USA), 100 U/ml penicillin, and 100 mg/ml streptomycin and cultured at 37˚C in humidified air with 5% CO_2_. Specially, the human monocytic THP-1 cells were maintained in RPMI 1640 culture medium containing 10% FBS and supplemented with 10mM 4-(2-hydroxyethyl)-1-piperazineethanesulfonic acid (HEPES; GIBCO-BRL; Invitrogen, CA, USA), 1mM pyruvate (GIBCO-BRL; Invitrogen, CA, USA), 2.5 g/l D-glucose (Merck, Darmstadt, Germany) and 50pM β-mercaptoethanol (GIBCO-BRL; Invitrogen, CA, USA). Human THP-1 monocytes were differentiated into macrophages using 200ng/ml PMA (phorbol-12-myristate-13-acetate) for 48 hours. Once differentiated (M0 macrophages), the adhered cells were further incubated with 20ng/ml interferon-γ (IFN-γ) and 10pg/ml lipopolysaccharide (LPS) during 72 hours in order to obtain M1-polarized macrophages, or with 20ng/ml IL-4 and 20ng/ml IL-13 during 72 hours for M2-polarized macrophages. In the co-culture experiments, pancreatic cancer cells (5 × 10^5^ cells/well) were added into the lower chamber of a 6-well transwell apparatus with 0.4 μm pore size (Costar, Cambridge, MA). Macrophages with different polarization were added into the upper chamber. Selected co-cultures were treated with neutralizing antibodies to CCL18 (Cat. No. ab9849, Abcam, Cambridge, MA) at 10 μg/ml.

**RNA extraction and quantitative real-time PCR (qRT-PCR)**

Total RNA was extracted from tissues or cultured cell lines using TRIzol reagent (Invitrogen, San Diego, CA, USA) following the manufacturer's instructions. RNA concentration was measured with NanoDrop ND-2000 spectrophotometers (Life Technologies, CA, USA) and then the total RNA (500 ng) was then transcribed to cDNA in a final volume of 10 μl using oligodT primers and SuperScript II reverse transcriptase (Invitrogen, San Diego, CA, USA). Real-time PCR was performed using SYBR Green reaction mix (Qiagen, Germany) and analyzed on a Roche Light-Cycler system (Roche, Basel, Switzerland). The results were normalized to the expression of β-actin. All the primer sequences were listed in Table S1. The qRT-PCR data were analyzed and expressed relative to CT (cycle threshold) values. The qRT-PCR results were analyzed and showed as the fold change (2^-∆∆CT^). For expressions in tissues, the levels were firstly normalized to β-actin expression as △CT and then compared with one of the tissues and converted to the fold change (2^-∆∆CT^). For the analysis of relative gene expressions in tissues or cells, the levels were compared with the controls and converted to the fold change (2^-∆∆CT^). The quantitative PCR reaction for each sample was repeated in triplicate.

**Cell transfection and viral infection**

All the small interfering RNAs (siRNAs) been used at the study were as follows: PITPNM3 siRNA (si-PITPNM3), VCAM-1 siRNA (si-VCAM-1) and scrambled siRNA (si-NC) were gained from GenePharma Co. (Shanghai, China). The siRNAs were transfected into cells using Lipofectamine 3000 (Life Technologies, CA, USA) according to the manufacturer’s instruction. After 48 hours, the efficiency of siRNA knockdown was confirmed via qRT-PCR and Western blot analysis. Stable human VCAM-1 knockdown PANC-1 cells were generated using two independent shRNAs in the lentiviral vector (pLKO.1-puro vector) as described in Table S2. 72 hours after transfection of 293T cells, the viral supernatants were collected. Using a LentiX™ Concentrator overnight at 4°C (Clontech, Mountain View, CA, USA), lentiviral particles were concentrated and titered to 10^9^ TU/ml (transfection unit/ml). PANC-1 cells (5 × 10^5^ cells/well) were seeded in 6-well culture plates and maintained in DMEM with 10% FBS and then infected with virus and polybrene 24 hours later. Positive clones were screened with puromycin (2μg/ml and 5μg/ml, respectively) for 2-3 weeks to establish the following new stable cell lines: PANC-1-sh-VCAM-1 (VCAM-1 stable knockdown) and PANC-1-sh-NC (negative control). All oligonucleotide sequences were listed in Table S2.

**Cell proliferation assay and colony formation assay**

Cell proliferation was measured using a cell counting kit-8 (CCK-8) assay according to the manufacturer’s instructions. Cells were seeded in 96-wells plates (2 × 10^3^ cells/well), at the appropriate time (0, 24, 48, 72 and 96 hours), CCK-8 solution (10 μL, Dojindo Molecular Technologies, Kyushu, Japan) was added and the cells were incubated for 4 hours at 37^o^C. Absorbance was measured at a wavelength of 450 nm. The assays were repeated three times.

For colony formation assay, a total of 600 cells transfected with si-VCAM-1 or si-NC were seeded in 6-well plates and maintained in media containing 10% FBS at 37^o^C, which was replaced every 3 days. After 14 days, colonies were fixed with methanol and stained with 0.1% crystal violet ([Sigma-Aldrich](http://www.sigmaaldrich.com/catalog/product/sigma/ht90132?lang=en&region=US), Milwaukee, USA). Visible colonies were then manually counted. Wells were measured in triplicate for each treatment group.

**Cell apoptosis and cell-cycle analysis**

For cell apoptosis, PANC-1 and Capan-2 cells (5 × 10^4^ cells) were collected via trypsin digestion, washed with cold phosphate-buffered saline (PBS), and resuspended in Annexin V binding buffer. AV-FITC（Becton Dickinson Biosciences, USA）at a final concentration of 1 µg/ml and 250 ng of PI were added to a mixture containing 100µL each of cell resuspension and binding buffer (Becton Dickinson Biosciences, USA). The mixture was incubated in the dark for 15 minutes at room temperature. Cells were washed once with binding buffer and resuspended in 400 µL of binding buffer prior to flow cytometric analysis using a BD FAC Scan Flow Cytometer (BD, Mountain View, USA).

For cell-cycle analysis, PANC-1 and Capan-2 cells (5 × 10^4^ cells) were collected and washed three times with PBS. Cells were then incubated in propidium iodide (PI) staining solution (RNase A 100 ug/mL and PI 500 ug/mL) for 30 minutes at 4 °C, and cells were analyzed by flow cytometry. Each study was repeated at least three times.

**Flow cytometry for detecting CD68 and CD206**

Cells were stained with fluorochrome-conjugated human monoclonal antibodies against CD68-PE (Cat.No. 85-12-0689-42, eBioscience), CD206-FITC (Cat.No. 12-2069, eBioscience) according to the manufacturer’s instructions, and they were subsequently analyzed by multicolor flow cytometry using BD FAC Scan Flow Cytometer (BD, Mountain View, USA).

**Wound healing assay and transwell assay**

Cells were incubated with normal cell growth medium in 6-well plates. Once cultures reached 85% confluency, the cell layer was scratched with a 10 μl sterile pipette tip and washed with culture medium, then exchanged with medium containing 1% FBS cultured for 48 hours. To prevent cell proliferation, which could confound the analysis of cell migration into the wound, cells were preincubated with mitomycin C (10 μg/ml) for 1 hour at 37 °C. At different two points (0 hour, 32 hours), images of the plates were acquired using a microscope. Experiments were performed at least three times.

Cell migration and invasion assays were performed using transwell chambers (24-well insert, 8 μm, Corning Costar Corp). Cells were harvested at 48 hours after transfection and then collected. 5 × 10^4^ cells were suspended in serum-free medium and placed in the uncoated (migration assay) or 1:8 diluted Matrigel-coated (invasion assay, BD Biosciences, NJ, USA) upper chamber. The lower chamber was filled with 500 ul of medium containing 20% FBS. After incubation for 36 hours at 5% CO_2_ at 37°C, the cells remaining on the upper chamber were removed with cotton wool, cells migrating to the bottom surface of the membrane from the upper chamber were fixed with methanol, stained with 0.1% crystal violet solution, then were imaged and five random fields were counted. Experiments were performed at least three times.

**Immunofluorescence (IF) analysis**

Cells were cultured to confluency on uncoated glass cover slips for 24 hours, and fixed in 4% paraformaldehyde at room temperature for 15 minutes. After washing with PBS, the adherent cell monolayer was permeablized with 0.1% Triton X-100 in PBS and blocked for 1 hour with 10% donkey serum in 1% BSA, followed by overnight incubation at 4 °C with primary antibodies: P65 (Cat.No. 6956, Cell Signaling Technology, Beverly, MA). After washing with PBS, cells were incubated with the Alexa Fluor 488 donkey anti-mouse IgG (H+L) (Molecular Probes, Eugene, OR) for 1 hour. Cells were then washed three times with PBS and mounted in Vectashield mounting medium containing DAPI (#ab104139, Abcam). The slides were analyzed using a confocal laser scan microscope.

**Western blotting analysis**

Cells were washed in PBS and lysed using the protein extraction reagent RIPA (Invitrogen, Carlsbad, CA) supplemented with a protease inhibitor cocktail (Roche, Pleasanton, CA, USA) and PMSF (Roche). The protein concentration was calculated using a bicinchoninic acid protein assay kit (Pierce, Rockford, IL, USA). Equivalent amounts of proteins (35 μg) from each sample were electrophoresed on a 8% SDS-polyacrylamide gel (SDS-PAGE) and then transferred to a polyvinylidene fluoride membrane, blocked in 5% fat-free milk for 2 hours at room temperature, and incubated with the following specific primary antibodies: rabbit anti-human VCAM-1 antibody (1:1000, #ab134047, Abcam), rabbit anti-human t-p65 antibody (1:800, #8242, Cell Signaling Technology (CST), Boston, USA), rabbit anti-human p-p65 antibody (1:800, #3033, CST, Boston, USA), rabbit anti-human IKKα antibody (1:1000, #2682, CST, Boston, USA), rabbit anti-human p-IKK antibody (1:1000, #2697, CST, Boston, USA), rabbit anti-human IKBα antibody (1:800, #4814, CST, Boston, USA), rabbit anti-human p-IKBα antibody (1:800, #2859, CST, Boston, USA), rabbit anti-human β-actin antibody (1:2000, #ab8227, Abcam) or rabbit anti-human GAPDH antibody (1:2000, #ab18162, Abcam). GAPDH or β-actin was used as a loading control. HRP-linked secondary antibody is goat anti-rabbit IgG (1: 5000; CST, Boston, USA). An ECL chemiluminescence kit (Pierce) was used to detect specific bands and autoradiograms were quantified by densitometry (Quantity One software, Bio-Rad, Hercules, CA, USA) using GAPDH or β-actin as a control.

**ELISA assay**

A standardized procedure for the sandwich ELISA was established after optimization of experimental parameters. CCL18 ELISA kits were from R&D system (Minneapolis, MN, USA). CCL22 ELISA kits were from RayBiotech (Norcross, Ga). IL-10 ELISA kits were from RayBiotech (Norcross, Ga). Briefly, monoclonal antibodies were coated onto standard ELISA plates (100 ml/well) overnight at 4℃. After gently washed with PBS, uncoated sites were then blocked by 100 ml 10% FBS in PBS for 1 hour at room temperature. Duplicates of samples were gently added and the whole plates were incubated for 1 hour. After gently washed with PBS, the plates were incubated with biotinylated antibody against CCL18, CCL22 or IL-10, for 2 hours at room temperature. Subsequently, avidin-conjugated horseradish peroxidase was gently added for 30 minutes. 100 ml of tetramethylbenzidine (TMB) was used to performed colour reaction for 30 minutes and the reaction was then terminated with 2 M sulfuric acid. All plates were immediately read at 450 nm using an ELISA reader. The protein concentrations were quantified by extrapolation from the standard curve.

**Immunohistochemistry staining and scoring**

Paraffin-embedded samples of primary carcinomas were stained for VCAM-1 or CCL18. Sections were deparaffinized in xylene and rehydrated in a graded series of ethanol, followed by heat-induced epitope retrieval in citrate buffer (pH = 6.0). Antigen retrieval was performed in 10 mmol/L citrate buffer (pH = 6.0) in a microwave oven for 15 minutes. The activity of endogenous peroxidases was blocked by the addition of 3% hydrogen peroxide for 10 minutes at room temperature. Rabbit anti-human VCAM-1 antibody (1:200, #ab134047, Abcam), rabbit anti-human CCL18 antibody (1:30, MAB394, R&D), rabbit anti-human PITPNM3 antibody (1:50, Santa Cruz Biotechnology, Santa Cruz, CA) and Ki-67 (1:500, #ab6526, Abcam) was applied overnight at 4 °C, and after washing three times in PBS, sections were immunostained with a goat anti-rabbit IgG (1: 5000; CST, Boston, USA) for 1 hour at 37 °C. The slides were incubated with streptavidin-HRP conjugate complex for 45 minutes at 37 °C. After rinsing three times in PBS, the sections were developed with 3, 3’-diaminobenzidine. Sections were counterstained with hematoxylin. Sections of skin tissues known to stain positive for VCAM-1 or CCL18 were used as positive controls, and normal goat serum and PBS substituting the primary antibody were used as negative controls.

The expression levels of CCL18 and VCAM-1 were scored semiquantitatively based on staining intensity and distribution using the immunoreactive score (IRS) as described elsewhere.^37,38^ Briefly, immunoreactive score (IRS) = SI (staining intensity) × PP (percentage of positive cells). SI was assigned as: 0 = negative; 1 = weak; 2 = moderate; 3 = strong. The percentage of positive tumor cells were calculated after at least 10 view fields at 400 × magnification were counted per section. PP is defined as 0 = 0%; 1 = 0-25%; 2 = 25-50%; 3 = 50-75%; 4 = 75-100%. For categorization of the continuous VCAM-1 and CCL18 values into low, moderate and high, we chose a commonly used cutoff point for the measurements (total score range 0-12: low, cut point of 0-2; moderate, cut point of 3-6; high, cut point of 8-12). Staining was professionally assessed by two pathologists based on the scoring criteria. Cases with discrepancies were jointly reevaluated until a consensus was reached.

**Preparation of conditioned medium**

Pancreatic cancer cells were grown to about 80% confluence in growth culture medium. Following washes in DMEM medium (GIBCO-BRL; Invitrogen, CA, USA) or RPMI 1640 (GIBCO-BRL; Invitrogen, CA, USA), cells were incubated for 24 hours in DMEM medium or RPMI 1640 supplemented with 10% fetal bovine serum (FBS; GIBCO-BRL; Invitrogen, CA, USA), 100 U/ml penicillin, and 100 mg/ml streptomycin and cultured at 37 ˚C in humidified air with 5% CO_2_. Medium conditioned by pancreatic cancer cells was then harvested, centrifuged at 2500g for 5 minutes and filtrated by 0.22 μm filters to eliminate cell debris.

**Tumor formation assay in a nude mouse model**

The athymic BALB/c nude mice aged from 4-6 weeks were purchased and maintained at the Laboratory Animal Center of Sun Yat-sen University in a specific pathogen-free environment. Mice were given continuous access of food and water. The animal care and experimental protocols were approved by the institutional guidelines of Guangdong Province and by the Use Committee for Animal Care. PANC-1 cells stably transfected with sh-VCAM-1 or sh-NC were cultured in six well plates for 48 hours. Then, the cells were collected, washed with phosphate-buffered saline and resuspended at 1 × 10^8^ cells/ml. A total of 100 μl of suspended cells with stable knockdown of VCAM-1, or mock cells, were respectively injected subcutaneously into left and right bilateral hind leg of mice. At day 3 after the injection of tumor cells, the tumor growth was evaluated once every 3 days by measuring the length and the width with electronic calipers. The tumor volume was calculated using the following formula: V = (L × W^2^) / 2 (V, volume; L, length diameter; W, width diameter). The mice were sacrificed by cervical dislocation at 27 days post injection, and tumors were collected for further study (weight measurement and RNA extraction). VCAM-1 levels were determined by qRT-PCR and western blotting analysis.
