# Supplementary material for: Tumor-associated macrophages promote progression and the Warburg effect via CCL18/NF-kB/VCAM-1 pathway in pancreatic ductal adenocarcinoma
Source: Cell Death Dis. 2018 Apr 18;9(5):453. doi: 10.1038/s41419-018-0486-0 (PMC5906621; doi:10.1038/s41419-018-0486-0)
Supplement: Supplementary file 8 — Supplementary figure legends [file 41419_2018_486_MOESM8_ESM.docx]

**Figure S1.** **Infiltration of CCL18-positive TAMs in pancreatic cancer and characterization of THP-1-derived M2 macrophages.** (A) Representative images of CCL18 staining in PDAC tissues and corresponding adjacent non-tumorous tissues by immunohistochemistry analysis (magnification: ×100, × 200 and × 400). (B) Typical morphology of THP-1 monocytes-derived macrophages by light microscopy (×100 and × 200). (C) Surface marker detection of CD68 and CD206 by flow cytometry in IL-4 and IL-13-activated THP-1 derived macrophages. (D-I) THP-1 macrophages were incubated with IL-4 and IL-13 for 24, 48 or 72 hours, and mRNA expression of M2 macrophage markers was studied by RT-PCR. (J-L) The CCL18, CCL22 and IL-10 secretion in the culture medium by macrophages was measured by ELISA assay. *, ** or ***: significantly different from the corresponding control, *p* < 0.05, *p* < 0.01 or *p* < 0.001, respectively. #, ## or ###: significantly different, *p* < 0.05, *p* < 0.01 or *p* < 0.001, respectively.

**Figure S2.** **CCL18/PITPNM3 axis plays a key role in the crosstalk between TAMs and pancreatic cancer cells.** (A) The ratio of a panel of cytokine mRNA in IL-4-activated THP-1-derived macrophages versus that in unactivated THP-1-derived macrophages, as assayed by qRT-PCR. (B-C) CCL18 mRNA and protein levels in pancreatic cancer cells alone or treated with 20ng/ml IL-4, as assayed by qRT-PCR and ELISA assay. The unactivated THP-1-derived macrophages were used as the positive control. (D) The qRT-PCR demonstrated that the expression of CCL18 is increased in PDAC tissues compared with normal pancreatic tissues. (E) As determined by ELISA assay, serum CCL18 levels are increased in the PDAC patients compared with patients with benign pancreatic disease. (F) The mRNA level of four potential CCL18 receptors in four pancreatic cancer cell lines, as assayed by qRT-PCR. (G) The qRT-PCR demonstrated that the expression of PITPNM3 is increased in PDAC tissues compared with normal pancreatic tissues. (H) Immunohistochemical staining shows that both CCL18 and its corresponding receptor PITPNM3 are elevated in pancreatic cancer tissues compared with normal pancreatic tissues. ns.: not significantly different. *, ** or ***: significantly different from the corresponding control, *p* < 0.05, *p* < 0.01 or *p* < 0.001, respectively.

**Figure S3.** **TAMs upregulates VCAM-1 expression in pancreatic cancer cells via CCL18/PITPNM3/NF-kB signalling pathway.** (A-B) The mRNA and protein levels of VCAM-1 in pancreatic cancer cells as determined by qRT-PCR and western blotting analysis. The Capan-2 pancreatic cancer cells were cultured alone or were treated with 20 ng/ml CCL18 for 24 hours, or the si-PITPNM3 Capan-2 cells were subsequently treated with 20 ng/ml CCL18 for 24 hours. The anti-CCL18 neutralizing antibody (10 μg/ml) was added into the experiment simultaneously as the Capan-2 cells were cocultured with TAMs. The Capan-2 pancreatic cancer cells without any treatment were used as the control. (C) CCL18-induced phosphorylation of IKK, IKB and p65 can be reversed by PITPNM3 RNAi. Capan-2 cells were transfected with NC-siRNAs or PITPNM3-siRNAs for 48 hours and then treated with CCL18 for 24 hours. The cells were then prepared for detecting the indicated proteins by western blotting. (D-E) CCL18 induced upregulation of VCAM-1 mRNA and protein levels can be reversed by repressing NF-kB activation. BAY-117082 and JSH-23 are specific inhibitors of IKB and p65, respectively. (F) NF-kB activity in Capan-2 cells increases upon the treatment with CCL18 and TNF-a. NF-kB activity is exhibited by luciferase reporter assay. (G) Representative immunofluorescence photos showed that CCL18 induced p65 translocation in Capan-2 cells can be reversed by silencing the CCL18 receptor PITPNM3 or by NF-kB pathway inhibitors (BAY-117082 and JSH-23). ns.: not significantly different. ** or ***: significantly different from the corresponding control, *p* < 0.01 or *p* < 0.001, respectively.

**Figure S4.** **VCAM-1 on pancreatic cancer cells binds THP-1 monocytic cells *in vitro*.** (A-B) Hoechst33342 (blue)-labelled PANC-1 or Capan-2 monolayers were incubated with a suspension of BCECF-AM (green)-labelled THP-1 cells, a human monocyte cell line. Representative photomicrographs of adherent BCECF-AM-labelled THP-1 cells on the monolayer of indicated PANC-1 or Capan-2 cells transfected with si-NC or si-VCAM-1 (magnification: × 200). (C) Quantification of the adherent BCECF-AM-labelled THP-1 cells to PANC-1 or Capan-2 cells transfected with si-NC or si-VCAM-1 at magnification of 200. (D) Quantification of the adherent BCECF-AM-labelled THP-1 cells to PANC-1 or Capan-2 cells treated with anti-IgG or anti-VCAM-1 at magnification of 200. (E-F) Addition of a blocking antibody against VCAM-1 significantly inhibited the binding of THP-1 cells to PANC-1 or Capan-2 cells (magnification: × 200). ***: significantly different from the corresponding control, *p* < 0.001, by Student’s *t*-test.
